# Prevalence and characterisation of energy drink consumption in Europe: a systematic review

**DOI:** 10.1017/S1368980025100463

**Published:** 2025-06-03

**Authors:** Ana Teijeiro, Nerea Mourino, Guadalupe García, Cristina Candal-Pedreira, Julia Rey-Brandariz, Carla Guerra-Tort, Marta Mascareñas-García, Agustín Montes-Martínez, Leonor Varela-Lema, Mónica Pérez-Ríos

**Affiliations:** 1 Department of Preventive Medicine and Public Health, Universidade de Santiago de Compostela, Santiago de Compostela, Galicia, Spain; 2 University of A Coruña, University College of Nursing, Oza, 15071 A Coruña, Spain; 3 CIBER de Epidemiología y Salud Pública, Centro de Investigación Biomédica en Red Epidemiología y Salud Pública, Madrid, Spain; 4 Epidemiology, Public Health and Health Services Evaluation Group, Instituto de Investigación Sanitaria (IDIS), University of Santiago de Compostela, Santiago de Compostela, Galicia, Spain

**Keywords:** Systematic review, Energy drinks, Caffeine, Prevalence, Europe, Population characteristics

## Abstract

**Objective:**

Energy drinks (ED) can cause cardiovascular, gastrointestinal and other health disorders. These effects are particularly pronounced in youth. The aim of this study was to systematically review the literature on the consumption of ED in European countries.

**Design:**

A systematic bibliographic search was performed in November 2024 in EMBASE, MEDLINE (Ovid), Scopus and Cochrane databases with no restrictions on country, study period, study design and language.

**Setting:**

ED are beverages high in caffeine, sugar and other stimulants.

**Participants:**

A total of 2008 studies were identified and reviewed by four researchers. Ninety-four met the inclusion criteria and were extracted in a table designed ad hoc.

**Results:**

The included studies showed differences regarding their design, definition of consumption and time frame under study. The most studied frequency of ED consumption was weekly consumption, and the most studied population was school students. An increase in the prevalence of consumption was observed when tracking ED consumption over time. Variables most related to consumption were low socio-economic status, alcohol and tobacco consumption, physical activity, age and sex.

**Conclusions:**

It is difficult to have a clear picture of the extent of ED consumption in Europe, mainly due to differences in the design of the studies and the lack of periodicity of the estimates in different countries. However, given the health problems that have been associated with ED consumption, regulation of these beverages is essential, especially in youth.

Energy drinks (ED) are generally defined as beverages with a high content of caffeine, sugar and other stimulant substances such as taurine, guarana, ginseng or vitamins^(1)^. However, there is no general consensus on their definition. The European Commission Scientific Committee on Food defines ED as a customary commercial name for beverages that contain high levels of caffeine together with ingredients not commonly found in sodas and juices^(2)^.

The discrepancies in the definition of ED are mainly due to the variation in their composition and the lack of rigor in their labelling. In general, most brands claim to contain between 70 and 80 mg of caffeine. On the other hand, the caffeine contained in other ingredients like guarana, which may contain up to 40–80 mg per gram, might not be declared on labels because it is not mandatory^(3)^. It should be noted that the European Food Safety Authority recommends that the maximum daily intake of caffeine should not exceed 400 mg^(4)^.

Control of caffeine intake is critical due to its multiple adverse effects. Caffeine can cause cardiovascular problems such as arrhythmia, especially tachycardia; gastrointestinal disturbances such as nausea and vomiting and neuropsychiatric effects such as psychomotor agitation, insomnia and anxiety, among others^(5)^. Excess ingredients such as sugar may also lead to complications like obesity or diabetes^(6)^.

In 2021, the European Food Safety Authority estimated the prevalence of ED consumption in sixteen European countries. The results revealed that while 30 % of adults had consumed ED in the last year, the prevalence among adolescents during the same period reached 68 %^(7)^. Young consumers often perceive these beverages as cool, a perception that is largely influenced by marketing campaigns linking ED with extreme sports^(5,8–15)^. Majori et al.^(16)^ define this consumption as a social phenomenon. Concerns regarding high underage consumption have prompted regulatory actions in some countries, such as Lithuania and Latvia, which have instituted regulations governing their sale^(17)^. Similarly, Canada has required manufacturers to comply with caffeine limits, marketing restrictions and health risk warnings^(18)^. Other European countries such as Poland^(19)^ and Norway^(20)^ are also considering following the steps of Lithuania and Latvia and implementing measures to prohibit the sale of ED to minors under the age of 16.

In order to design appropriate regulatory policies, it is necessary to acknowledge the present situation of the product to be regulated. For this reason, the aim of this study was to systematically review the literature on the consumption of ED in European countries and the characterisation of the consumers.

## Methods

A systematic review was conducted following the PRISMA 2020 (Preferred Reporting Items for Systematic Reviews and Meta-Analyses) guidelines^(21)^. The systematic review protocol, registered with number CRD42023473014 in the PROSPERO database, outlined a comprehensive analysis of the worldwide prevalence of ED consumption.

The studies were meticulously organised by continent to streamline the management of the voluminous information and enable a comprehensive examination of country-level variations within each continent. This categorisation enhanced the analytic precision and provided a more profound comprehension of regional disparities in ED consumption. Here, we only reviewed the studies in Europe.

Original articles that fulfilled PECOS (Population, Exposure, Comparator, Outcome and Study design) criteria (Table 1) were selected considering the following question: ‘What is the prevalence of ED consumption in Europe and the characteristics of their consumers?’.

**Table 1.** PECOS criteria

**Population**	European population
**Exposure**	Energy drink consumption
**Comparator**	Not applicable
**Outcome**	Prevalence of energy drink consumption and consumers characterisation
**Study design**	All except systematic reviews

### Search strategy

A bibliographic search was performed in July 2023 in EMBASE, MEDLINE (Ovid), Scopus and Cochrane databases and later updated in November 2024, after applying a pre-designed search strategy drawn up by three expert reviewers (ATT, NMC, MPR) (see online supplementary material, Supplemental Table 1).

The search strategy was developed by combining MeSH terms, emtree terms and free terms. The MeSH and emtree terms were ‘Energy drinks’, ‘Prevalence’ and ‘Epidemiology’. The free terms were ‘drink*’, ‘beverag*’, ‘Caffein*’, ‘taurin*’, ‘consumer’ and ‘consumption’. No restrictions on country, study period, study design and language were applied for the bibliographic search.

Furthermore, the bibliographic references of selected articles were reviewed to ensure the inclusion of all possible studies.

The article with the earliest date of publication complying with the inclusion criteria set was considered the first publication on ED consumption.

### Inclusion/exclusion criteria

The review covered all studies which included prevalence data of ED consumption in any time frame, country and population, regardless of whether their main objective was the estimation of the prevalence of consumption.

Studies with the following characteristics were excluded: (a) studies that estimated the prevalence of overall caffeine consumption, without differentiating the type of beverage; (b) those that provided the combined prevalence of ED and other beverages and substances, such as alcohol, without providing individual ED outcomes; (c) those whose target population consisted exclusively of individuals with a range of medical conditions or substance dependence and (d) studies that covered more than one country without providing individual prevalences for each of them.

Moreover, studies published in languages different from Spanish, English or Portuguese, communications to conferences, letters, opinion articles, narrative reviews, systematic reviews with or without meta-analysis, simulation studies and retracted publications were excluded. Additionally, grey literature was not included because peer review was not guaranteed.

### Selection of studies and extraction of data

After eliminating duplicated references, four authors screened titles and abstracts of all studies yielded by the search through a blinded peer-review process. Subsequently, the same authors reviewed the full text of studies considered potentially relevant. Discrepancies, both in eligibility and in data extraction, were discussed and settled by consensus.

The four authors manually extracted data from the selected studies in a pre-designed extraction ad hoc sheet in Microsoft Excel, which was adapted to the STROBE checklist^(22)^. Discrepancies were discussed and settled by consensus.

Studies estimating ED consumption in European countries were selected for inclusion in this manuscript. For each study, information was extracted on:

a. Study characteristics: first author, year of publication, study period, country, study design, geographical scope (European, nationwide, regional or local), and, if applicable, the name of the study or survey from which the data were derived.

b. Population characteristics: sample size, population group (school students, university students or the general population, encompassing children, adolescents or adults), sex and age group, mean age or academic degree.

c. ED consumption data: definition of ED consumption, including the frequency of consumption (regular, occasional or infrequent) or periodicity (daily, weekly, monthly, yearly, ever in lifetime), the method used to ascertain consumption (including format of the questionnaire) and the prevalence of ED consumption, alone or mixed with alcohol. Prevalence of consumption was extracted only from studies that provided frequency data on ED consumption, both alone or mixed with alcohol. Both overall prevalence and prevalence by sex were included. Prevalence percentages stratified by age group or academic year were only included if overall prevalence was not available. When longitudinal studies estimated the prevalence of use over different time periods, the most recent year was extracted. In addition, if a study reported different intensity-based consumption percentages for the same time frame, the sum of all individual percentages was used to determine the overall prevalence for the specific period. For European studies with prevalence data from multiple countries, both global and country-specific prevalence percentages were included. If a study included the prevalence of ED consumption mixed with alcohol for different timeframes, that referring to the last month was included.

d. Characterisation of the ED consumers: dependent and independent variables conferring a higher likelihood of being an ED consumer were extracted. All models adjusted were considered, including those employing various definitions of ED consumers and those exploring sex differences and changes over time.

The results were structured in five sections: ‘Characteristics of the Studies and Population’, ‘Data Collection Methods’, ‘Prevalence of ED Consumption’, ‘Characterization of ED Consumers’ and ‘Evaluation of the Quality of the Studies’.

### Assessment of quality

Study quality was evaluated using an adaptation of the Newcastle–Ottawa scale^(23)^. The adaptation was made to better suit the nature of the methodological design of studies in our review, which were mainly cross-sectional. This adaptation allowed for a more accurate evaluation of key aspects, such as data collection methods, the definition and characterisation of ED consumption and the stratification of prevalence data, which are not fully addressed by the original scale, designed primarily for cohort and case–control studies.

Two authors screened each study separately evaluating the sample selection/strategy (representativeness of the sample, sample size and response rate between respondents and non-respondents), assessment of ED consumption (ascertainment, definition and characterisation of the ED consumption), comparability and outcome (stratification of the prevalence data on ED consumption, statistical test and assessment of potential biases/limitations) (see online supplementary material, Supplemental Table 2). Studies were blindly scored from 0 to 16 by each author, with the final score being reached by agreement. In case of any difference of opinion, a third author was consulted. Studies with a score ≤ 8 points were rated as poor quality, those with a score of 9–12 points as moderate quality and those with a score of ≥ 13 points as high quality.

The quality of the studies was analysed according to the range, mean and median of the score obtained with the Newcastle–Ottawa scale; and the factors which most contributed to the decrease in the score were identified.

## Results

### Characteristics of the studies and population

**Figure 1.**
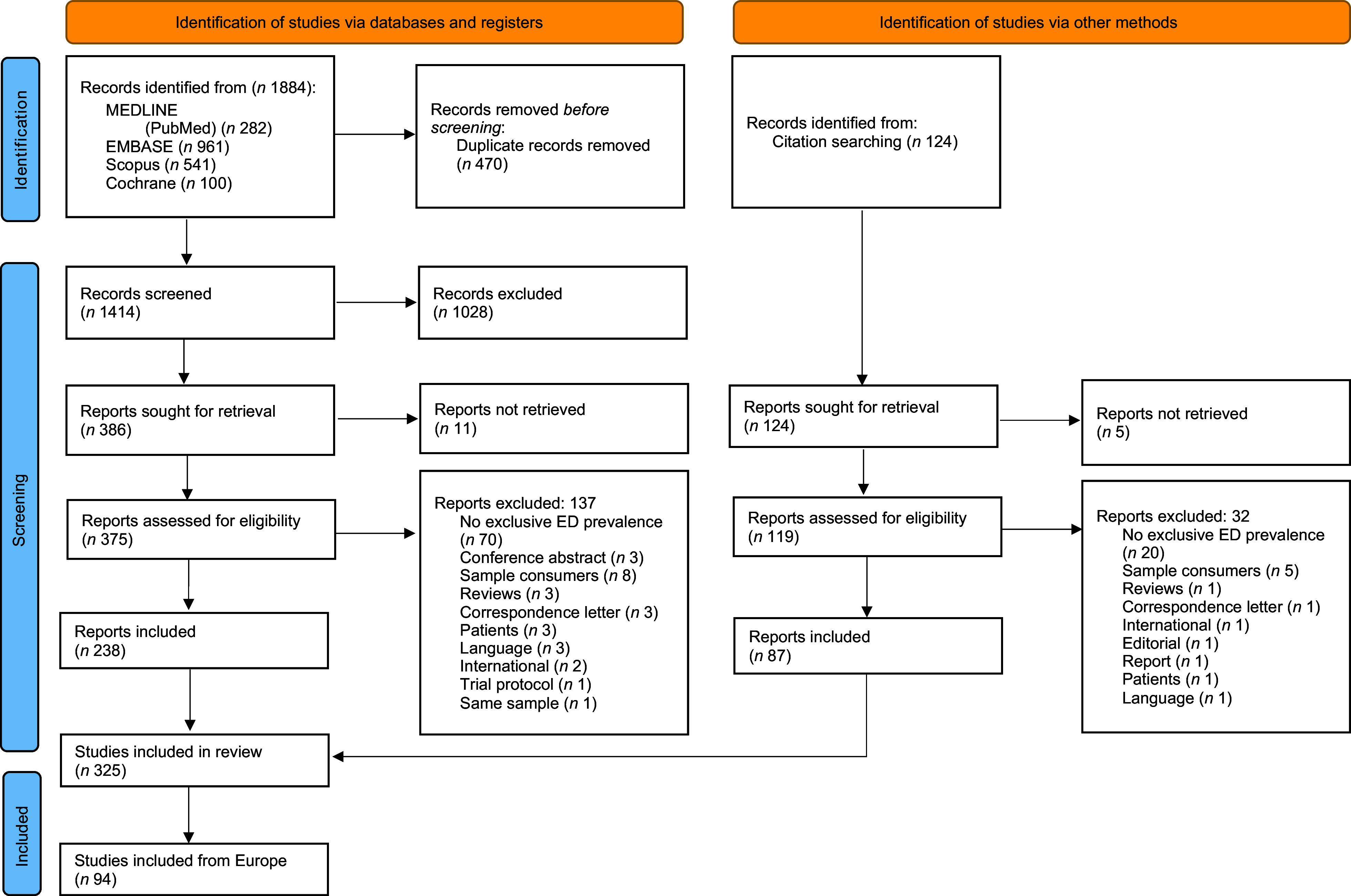
Flowchart of studies selected for the systematic review in accordance with the PRISMA 2020 guidelines.

The comprehensive literature search yielded 1884 studies, of which 470 were duplicates; 1028 were excluded for not meeting the criteria and eleven were not retrieved. 375 studies from the literature search and 119 from the review of bibliographic references (citation search) from the selected studies were deemed eligible for full-text review (Figure 1). 238 studies from the literature search and eighty-seven from the citation search estimated the prevalence of ED consumption worldwide. Therefore, a total of 325 were included. Of these, ninety-four were conducted in Europe during the period 2006–2023 (Table 2). The greatest number of studies, amounting to 19^(24–42)^, was published in 2021, indicating a notable increase in research output since the first publication in 2007. The studies spanned across twenty-seven European countries, with Italy (*n* 19), Poland (*n* 11) and Spain (*n* 9) emerging as the three countries with the highest volume of publications. Of the studies, the majority were conducted at the local and national levels (*n* 39 and 34, respectively), with one study at the European level, encompassing data from sixteen countries (Table 2).

**Table 2.** Study and population characteristics of the included studies (*n* 94)

Study characteristics										
Author	Publication year	Period	Country	Geographical scope	Design	Study/survey name	*n*	Population group	Age groups	Sex
Oteri A	2007	N/A	Italy	Cross-sectional	Local	N/A	450	University students	N/A	Both
Braun H	2009	2006–2007	Germany	Cross-sectional	Local	N/A	164	Adolescents and adults (athletes)	10–25	Both
Gambon DL	2011	N/A	The Netherlands	Cross-sectional	Local	N/A	502	School students	12–19	Both
James JE	2011	2009	Iceland	Cross-sectional	Nationwide	Youth in Iceland survey	7377	School students	8th and 10th graders	Both
Abreu AR	2013	N/A	Spain	Cross-sectional	Local	N/A	307	University students	18–30	Both
Gallimberti L	2013	2011–2012	Italy	Cross-sectional	Local	N/A	916	School students	11–13	Both
Kristjansson AL	2013	2012	Iceland	Cross-sectional	Nationwide	Youth in Iceland survey	3747	School students	15–16	Both
Maier LJ	2013	2012–2013	Switzerland	Cross-sectional	Local	N/A	6275	University students	17–68	Both
Zucconi S	2013	2012	Austria, Belgium, Cyprus, Czech Republic, Germany, Greece, Finland, France, Hungary, Italy, Poland, Romania, Spain, Sweden, The Netherlands and United Kingdom	Cross-sectional	European	Survey Nomisma-Areté for EFSA	52 000	Children, adolescents and adults	3–65	Both
Chuda A	2014	2013–2014	Poland	Cross-sectional	Local	N/A	131	University students	4th and 5th graders	Both
Flotta A	2014	2012	Italy	Cross-sectional	Regional	Youth Risk Behavior Survey	616	School students	15–19	Both
Friis K	2014	2010	Denmark	Cross-sectional	Regional	How are you? Survey	3923	Adolescents and adults	16–24	Both
Gornicka M	2014	2011–2012	Poland	Cross-sectional	Local	N/A	90	School students	15	Both
Kristjansson AL	2014	2013	Iceland	Cross-sectional	Nationwide	Youth in Iceland survey	11 132	School students	10–13	Both
Rudolph E	2014	N/A	Austria	Cross-sectional	Nationwide	N/A	700	Adolescents and adults	14–39	Both
Vilija M	2014	2012	Lithuania	Cross-sectional	Local	N/A	1747	School students	8th graders	Both
Casuccio A	2015	2012–2013	Italy	Cross-sectional	Local	N/A	794	University students	Mean: 21·5	Both
Gallimberti L	2015	2013–2014	Italy	Cross-sectional	Local	Project Pinocchio	1496	School students	5th, 6th, 7th and 8th graders	Both
Kristjansson AL	2015	2013	Iceland	Cross-sectional	Nationwide	Youth in Iceland survey	11 017	School students	16–20	Both
Liakoni E	2015	2014	Switzerland	Cross-sectional	Local	N/A	1139	School students	10–12th graders	Both
Nowak D	2015	2012–2013	Poland	Cross-sectional	Local	N/A	2629	School students	12–20	Both
Richards G	2015	2012–2013	United Kingdom	Cohort	Regional	Cornish Academic Project	2610	School students	12–16	Both
Barrense-Dias Y	2016	2012–2014	Switzerland	Cross-sectional	Local	ado@internet.ch survey	621	School students	14	Both
Cencek P	2016	N/A	Poland	Cross-sectional	Local	N/A	131	University students	19–25	Both
Koivusilta L	2016	2011	Finland	Cross-sectional	Regional	N/A	9446	School students	13	Both
Milovanovic DD	2016	NC	Serbia	Cross-sectional	Local	N/A	191	School students	18–19	Both
Pacifici R	2016	2013–2014	Italy	Cross-sectional	Regional	N/A	2621	Adolescents and adults	14–35	Both
Parezanovic GS	2016	N/A	Serbia	Cross-sectional	Local	N/A	258	University students	1th, 2th, 3th and 4th graders	Both
Treur JL	2016	2013–2014	The Netherlands	Cross-sectional	Nationwide	The Netherlands Twin Register	21 939	Adults	Mean: 40·8	Both
Vitiello V	2016	2014–2015	Italy	Cross-sectional	Regional	N/A	1007	University students	Mean: 22·7	Both
Wardenaar F	2016	2013	The Netherlands	Cross-sectional	Nationwide	DSSS*	1544	Adolescents and adults	15–80	Both
Wierzejska R	2016	2009–2010	Poland	Cross-sectional	Local	N/A	329	School students	11–13	Both
Casuccio A	2017	2014	Italy	Cross-sectional	Local	N/A	217	Adults	18–60	Both
Concerto C	2017	2013	Italy	Cross-sectional	Local	N/A	70	University students	25–40+	Both
Holubcikova J	2017	2014	Slovakia	Cross-sectional	Nationwide	HBSC**	8502	School students	11–15	Both
Holubcikova J	2017	2014	Slovakia	Cross-sectional	Nationwide	HBSC**	8977	School students	11–15	Both
Husarova D	2017	2014	Slovakia	Cross-sectional	Nationwide	HBSC**	7595	School students	11–15	Both
Scalese M	2017	2015	Italy	Cross-sectional	Nationwide	ESPAD***	30 588	School students	15–19	Both
Buja A	2018	2013–2014	Italy	Cross-sectional	Local	Project Pinocchio	1325	School students	11–13	Both
Degirmenci N	2018	2015–2016	Norway	Cross-sectional	Nationwide	Ungdata survey	31 091	School students	12–19	Both
Majori S	2018	2014–2015	Italy	Cross-sectional	Local	N/A	899	University students	18–39	Both
Martins A	2018	N/A	Portugal	Cross-sectional	Regional	N/A	1414	School students	11–17	Both
Scuri S	2018	2016–2017	Italy	Cross-sectional	Regional	N/A	1581	School students	N/A	Both
Thomas F	2018	2017	United Kingdom	Cross-sectional	Nationwide	N/A	3348	School students	11–19	Both
Totaro M	2018	2016–2017	Italy	Cross-sectional	Local	N/A	583	School students	14–18	Both
Galimov A	2019	2016–2018	Germany	Cross-sectional	Regional	N/A	6902–4529	School students	9–19	Both
Gruzieva TS	2019	2017–2019	Ukraine	Cross-sectional	Regional	N/A	948	University students	N/A	Both
Benkert R	2020	2011 and 2015	Switzerland	Longitudinal	Nationwide	Young adult survey Switzerland	2011: 10345/2015: 9761	Adults (military)	18–21	Male
Buja A	2020	2017	Italy	Cross-sectional	Nationwide	Gambling in Italy	15 602	School students	14–17	Both
Cruz Munoz V	2020	N/A	Spain	Cross-sectional	Local	BEENIS	4769	School students	13–18	Both
Holguín EP	2020	2018–2019	Spain	Cross-sectional	Local	N/A	353	University students	18–33	Both
Lebacq T	2020	2018	Belgium	Cross-sectional	Nationwide	N/A	10 289	School students	11–20	Both
Lehmann F	2020	2015–2017	Germany	Cross-sectional	Nationwide	EsKiMo II****	1353	School students	12–17	Both
Sljivo A	2020	2017	Bosnia	Cross-sectional	Local	N/A	812	University students	18–38	Both
Tanner T	2020	2010–2011	Finland	Cross-sectional	Nationwide	N/A	8537	Adults (military)	18–20	Male
Toth A	2020	2017	Hungary	Cross-sectional	Regional	N/A	631	High school and university students	Mean: 19	Both
Błaszczyk-Bębenek E	2021	2014–2015	Poland	Cross-sectional	Regional	N/A	508	School students	16–18	Both
Boleslawska I	2021	2020	Poland	Cross-sectional	Local	N/A	312	Adults	18–79	Both
Brumboiu I	2021	N/A	France and Romania	Cross-sectional	Local	N/A	1110	University students	18–25	Both
Dąbrowska-Galas M	2021	2019	Poland	Cross-sectional	Local	N/A	308	University students^†^	18–35	Both
Fernandes S	2021	2021	Portugal	Cross-sectional	Nationwide	N/A	1666	Adults	>18	Both
Franke AG	2021	2016	Germany	Cross-sectional	Local	N/A	683	University students and adults	Mean: 26·6	Both
Halldorsson TI	2021	2020	Iceland	Cross-sectional	Nationwide	Youth in Iceland survey	10 358	School students	13–15	Both
Jebrini T	2021	2020	Germany	Cross-sectional	Nationwide	N/A	2632	University students	N/A	Both
Kaldenbach S	2021	2017–2019	Norway	Longitudinal	Nationwide	Ungdata survey	278 891	School students	13–19	Both
Morgan K	2021	2013–2017	United Kingdom	Cross-sectional	Regional	SHRN*****-HBSC**	141 154	School students	11–16	Both
Oliver Angles A	2021	2015–2016	Spain	Cross-sectional	Regional	N/A	8078	School students	14–18	Both
Puupponen M	2021	2014 and 2018	Finland	Longitudinal	Nationwide	HBSC**	7405	School students	13 and 15	Both
Scalese M	2021	2008–2019	Italy	Longitudinal	Nationwide	ESPAD***	328 016	School students	15–19	Both
Schroder H	2021	N/A	Spain	Cross-sectional	Regional	BEENIS	3930	School students	13–18	Both
Svensson A	2021	2010 and 2011	Sweden	Longitudinal	Regional	Youth Health Development	1622	School students	N/A	Both
Vasic J	2021	2020–2021	Serbia	Cross-sectional	Local	N/A	184	School students (refugee population)	12–18	Both
Atienza-Carbonell B	2022	2020	Spain	Cross-sectional	Regional	N/A	1265	University students	Mean: 21	Both
Brunborg GS	2022	2017	Norway	Cross-sectional	Nationwide	MyLife	2916	School students	13–15	Both
Jeannou B	2022	2020	France	Cross-sectional	Regional	Perf-use-sports study	281	Adults (athletes)	Mean: 40	Both
Kaldenbach S	2022	2017	Norway	Cross-sectional	Regional	UngOpp	1353	School students	15–16	Both
Kosendiak AA	2022	2020	Poland	Cross-sectional	Nationwide	KomPAN	200	University students	19–21	Both
Riera-Sampol A	2022	2021	Spain	Cross-sectional	Local	N/A	886	University students	18–26	Both
Sammito S	2022	2020–2021	Germany	Cross-sectional	Nationwide	N/A	181	Adults (military)	24–59	Both
Soukiasian PD	2022	2018–2019	Greece	Cross-sectional	Nationwide	NUTSTUDY	28 491	Adults	>15	Both
Tomanic M	2022	2019–2020	Serbia	Cross-sectional	Local	N/A	1287	School students	15–19	Both
Erdös A	2023	2020–2021	Hungary	Cross-sectional	Local	N/A	180	University students	N/A	Both
Kaldenbach S	2023	2022	Norway	Cross-sectional	Nationwide	SHOT2022	53 226	University students	18–35	Both
Mititelu M	2023	2023	Romania	Cross-sectional	Nationwide	N/A	1754	Adults	>18	Both
Pavlovic N	2023	2018	Croatia	Cross-sectional	Local	N/A	424	University students	19–53	Both
PetrauskieneS	2023	2013–2014	Lithuania	Cross-sectional	Nationwide	N/A	1127	School students	14–16	Both
Protano C	2023	2021–2022	Italy	Cross-sectional	Nationwide	DiSCo	2165	University students	N/A	Both
Vogel C	2023	2008–2016	United Kingdom	Longitudinal	Nationwide	NDNS******	2587	School students	11–18	Both
Zivojinovic JI	2023	2022	Serbia	Cross-sectional	Local	N/A	431	University students	Mean: 24	Both
Carrasco-Garrido P	2024	2018–2019	Spain	Cross-sectional	Nationwide	ESTUDES	38 010	School students	14–18	Both
Di Martino G	2024	2023	Italy	Cross-sectional	Local	CCAP study	404	University students	Mean: 22	Both
Kostecha J	2024	2018, 2022 and 2023	Poland	Cross-sectional	Regional	N/A	2018: 491/ 2023: 539	School students	11–13	Both
Szepe O	2024	2023	Hungary	Cross-sectional	Local	N/A	561	University students	Mean: 24	Both
Vejrup K	2024	2022	Norway	Cross-sectional	Local	N/A	2225	Adults (military)	19–27	Both

*Dutch Sport nutrition and Supplement Study; **The Health Behaviour in School-aged Children study; ***European School Survey Project on Alcohol and Other Drugs; ****Eating study as a KiGGS Module; *****Student Health and Wellbeing; ******National Diet and Nutrition Survey.

† Those who had contraindicated doing sport were excluded.

Eighty-eight of the studies included were cross-sectional studies and six longitudinal (Table 2). Thirty-seven studies, all in school-aged populations, specified a name of a study or survey, with some sources commonly used across multiple studies. For instance, two Italian studies utilised data from the Pinocchio Project and another two from the European School Survey Project on Alcohol and Other Drugs (ESPAD). Two Spanish studies drew data from the BEENIS project. Additionally, one Finnish study and three Slovakian studies relied on data from the Health Behavior in School-aged Children (HBSC) project, while all four Icelandic studies were based on data from the Youth in Iceland Survey (Table 2).

The sample size of the included studies, covering a total of 1 247 135 children, adolescents and adults, ranged from 70^(43)^ to 328 016^(31)^ participants. Many studies had fewer than 1000 participants (40 of 94; 42·5 %). The majority of studies (77 out of 94) focused on school-aged children (aged 9–19 years) and university students (aged 18–53 years); notably, one study focused on refugee children^(34)^. The remaining studies included adult populations, either independently or in combination with children and adolescents. Two studies exclusively involved male military participants, one conducted in Switzerland^(44)^ and the other in Finland^(45)^ and two focused on athletes^(46,47)^ (Table 2).

### Data collection methods

Table 3 presents the definitions and methods used in each study to ascertain the prevalence of ED consumption. Variations were observed in terms of time frame and quantity, intensity or frequency of consumption. One study did not include any specific definition of ED consumption^(48)^. Five studies addressed consumption during specific contexts such as recreational activities, special occasions, exam periods and the COVID-19 pandemic^(3,26,35,49,50)^. Thirty-four studies specified in their definitions broader categories including ED, such as common food and drinks, dietary supplements and sport nutrition products, caffeinated beverages, unhealthy drinks, psychoactive substances, snacks and fast foods. Fifteen studies focused on specific brands, mainly Red Bull and Monster, and some studies investigated ED consumption alongside other psychoactive substances, notably alcohol (*n* 18).

**Table 3.** Study characteristics of energy drink (ED) consumption

Energy drink consumption characteristics						
Author	Publication year	Country	Population group	Main objective	Definition	Measure method
Oteri A	2007	Italy	University students	Yes	Use of almost 1 ED in the last month (alone or with alcohol) and the number of tins drunk daily and in the last month	Self-administered questionnaire (no further description)
Braun H	2009	Germany	Adolescents and adults (athletes)	No	Athletes’s use of a specific supplement, such as sports beverages (including sport drinks, ED and other drinks), in the 4 weeks preceding survey completion	Self-administered questionnaire via email
Gambon DL	2011	The Netherlands	School students	Yes	Consumption of beverages, such as ED, during the previous week	Self-administered questionnaire (no further description)
James JE	2011	Iceland	School students	No	Daily consumption of caffeinated beverages (including coffee, tea, cola drinks and ED: Red Bull or Magic Burn) and the number of cans/bottles or glasses drunk	Self-administered questionnaire supervised by teachers and monitored by a contact agent in the classroom
Gallimberti L	2013	Italy	School students	Yes	Ever use of ED and frequency in the last 2 months (once, about once a month/week, more or less every day)	Self-administered questionnaire administered by the research team
Kristjansson AL	2013	Iceland	School students	No	Daily consumption of caffeinated beverages (including coffee, tea, cola drinks and ED) and the number of glasses/cups drunk (<1 and ≥ 1)	Self-administered questionnaire supervised by teachers and monitored by a contact agent in the classroom
Maier LJ	2013	Switzerland	University students	No	Ever use of a list of substances including ED and frequency of use in the 30 d prior to their last exam	Self-administered online questionnaire
Ravelo AA	2013	Spain	University students	Yes	Ever and weekly consumption of ED (alone or with alcohol or other drugs), and the number of tins drunk weekly. It also considers consumption during exam periods and recreational activities, including partying.	Self-administered questionnaire (no further description)
Zucconi S	2013	Austria, Belgium, Cyprus, Czech Republic, Germany, Greece, Finland, France, Hungary, Italy, Poland, Romania, Spain, Sweden, The Netherlands and United Kingdom	Children, adolescents and adults	Yes	Consumption of ED (alone or with alcohol) at least once in the past year. Among adolescents, consumption during the last 3 days before the survey is also considered	Self-administered paper and online questionnaires for children and adolescents, and mixed-mode survey (CAWI: Computer-Assisted Web Interview and CATI: Computer Assisted Telephone Interview) for adults
Chuda A	2014	Poland	University students	Yes	Frequency of ED consumption (occasionally, daily, few times a week and several times a month). It also considers consumption during exam sessions	Self-administered online questionnaire
Flotta A	2014	Italy	School students	Yes	Ever use of ED (alone or with alcohol) during life and frequency in the previous 30 d	Self-administered questionnaire (printed form)
Friis K	2014	Denmark	Adolescents and adults	Yes	ED consumption (Red Bull, Cult and Burn) considering different frequencies: more than once; 5–7 times per week; 3–4 times per week; 1–2 times per week; rarely/never	Self-administered questionnaire (printed form by postal mail)
Gornicka M	2014	Poland	School students and university students	No	Consumption of caffeine-containing beverages, such as coffee, colas and ED	Self-administered questionnaire (no further description)
Kristjansson AL	2014	Iceland	School students	No	Daily consumption of caffeinated beverages (including cola drinks: Cocacola, Pepsi Cola; and ED: Red Bull, Magic Burn, Monster, Xl) and the number of cans/bottles or glasses drunk	Self-administered questionnaire supervised by teachers in the classroom
Rudolph E	2014	Austria	Adolescents and adults	No	Consumption in the past 12 months of caffeine-containing medicines, foods and beverages, such as chocolate, coffee, colas, teas, ED and energy shots	Face-to-face questionnaire by a professional market research company
Vilija M	2014	Lithuania	School students	No	Daily consumption of ‘unhealthy’ beverages such as sugar-sweetened/soft (cocacola, Pepsi, Fanta, etc.), ED and sports drinks (Red bull, Energiser, etc.), coffee, spirits (vodka, brandy, liquors), light alcoholic drinks (beer, wine, cider) and flavoured milk	Self-administered questionnaire after receiving detailed information from school nurse
Casuccio A	2015	Italy	University students	Yes	Current regular ED (sugared or sugar-free) consumption (alone or with alcohol), and the number of cans usually drunk. It also considers frequency of consumption (every day, 3–5 d per week, 2–4 times a month, ≤ 1 time a month) and side effects after consumption	Self-administered questionnaire in the classroom
Gallimberti L	2015	Italy	School students	Yes	Ever use of ED in the past month. It also considers dual use with alcohol in the last 3 months	Self-administered questionnaire administered by research team
Kristjansson AL	2015	Iceland	School students	No	Daily consumption of caffeinated beverages (including coffee, tea, cola drinks and ED), and the number of glasses/cups drunk (1 and ≥ 2). It also considers ever dual consumption with alcohol (once and ≥ 2 times)	Self-administered questionnaire supervised by teachers and monitored by a contact agent in the classroom
Liakoni E	2015	Switzerland	School students	No	Ever use of one or more prescription drugs without having a medical indication, recreational drugs or soft enhancers. Soft enhancers included energy drinks. Last year and last month used as cognitive enhancement	Self-administered online questionnaire supervised by teachers in the classroom
Nowak D	2015	Poland	School students	Yes	Consumption of ED (alone or mixed with alcohol) considering different frequencies (every day, few times a week, once a week and once a year), and the amount in terms of the number of 250 ml cans/bottles	Self-administered questionnaire in the classroom
Richards G	2015	United Kingdom	School students	Yes	Frequency (once a months, once/twice a week, most days and every day) of common foods and drinks (including ED, cola, coffee and tea)	Self-administered questionnaire in the classroom delivered by the respective academies
Barrense-Dias Y	2016	Switzerland	School students	Yes	Weekly consumption of ED	Self-completed online questionnaire (no further description)
Cencek P	2016	Poland	University students	Yes	Daily and ever consumption of ED. It also considers the dual use with other psychoactive substances, such as alcohol, cigarettes, designer drugs, coffee and medication	N/A
Koivusilta L	2016	Finland	School students	Yes	ED consumption considering different frequencies: once a week or less, approximately once a day and several times a day	Self-administered online questionnaire in computer classrooms
Milavanovic DD	2016	Serbia	School students	No	Experience with caffeine beverages consumption, such as coffee, tea, ED (Red Bull), soft drinks, chocolate and cocoa. It also considers the frequency of drinking and the amount consumed	Self-administered questionnaire (no further description)
Pacifici R	2016	Italy	Adolescents and adults	No	Ever use of psychoactive substances such as ED including frequency and type of beverage	Self-administered questionnaire (printed form) delivered at schools and recreational premises such as discos, pubs and night bars
Parezanovic GS	2016	Serbia	University students	Yes	Daily and weekly consumption of ED including types and quantities	Self-administered questionnaire in the classroom
Treur JL	2016	The Netherlands	Adults	Yes	Daily, weekly or (almost) never consumption of caffeinated coffee, tea, cola and ED	Self-administered questionnaire (no further description)
Vitiello V	2016	Italy	University students	Yes	Ever and weekly consumption of ED (alone or with alcohol)	Self-administered questionnaire (no further description)
Wardenaar F	2016	The Netherlands	Adolescents and adults	No	Use of dietary supplements and sport nutrition products (including sport drinks, such as ED and recovery drinks) in the last 12 months	Self-administered online questionnaire
Wierzejska R	2016	Poland	School students	Yes	Frequency of caffeine intake from cola beverages and ED (daily, several times a week and a month). It also considers the consumption of fast foods and salty snacks	Face-to-face questionnaire at school under a dietician’s supervision
Casuccio A	2017	Italy	Adults	Yes	Current regular ED (sugared or sugar-free) consumption (alone or with alcohol) and the number of cans usually drunk per week. It also considers frequency of consumption (3–5 d a week, 1–2 times a week, 2–4 times a month, ≤ 1 time a month)	Face-to-face questionnaire administered by two trained interviewers
Concerto C	2017	Italy	University students	No	Consumption of caffeinated drinks, such as coffee, tea, caffeinated soft drinks and ED during the previous week	Self-administered questionnaire (printed form)
Holubcikova J	2017	Slovakia	School students	Yes	Weekly consumption of ED, such as Red Bull (never, less than once a week, once a week, 2–4 d a week, 5–6 d a week, once a day, every day, more than once a day). It also considers regular dual use with alcohol	Self-administered questionnaire distributed by trained research assistants in the classroom
Holubcikova J	2017	Slovakia	School students	Yes	Regular or chronic consumption of ED, such as Red Bull (once a week and more)	Self-administered questionnaire distributed by trained research assistants in the classroom
Husarova D	2017	Slovakia	School students	Yes	Weekly consumption of ED, such as Red Bull (never, less than once a week, once a week, 2–4 d a week, 5–6 d a week, once a day, every day, more than once a day)	Self-administered questionnaire distributed by trained research assistants in the classroom
Scalese M	2017	Italy	School students	Yes	ED use (alone or with alcohol) in the last 12 months	Self-administered questionnaire (no further description)
Buja A	2018	Italy	School students	No	Psychoactive substance use at least once in the last month (alcohol, ED and tobacco)	Self-administered questionnaire administered by research team
Degirmenci N	2018	Norway	School students	Yes	Daily to once a month or less consumption of ED	Self-administered online questionnaire in the classroom with the teacher
Majori S	2018	Italy	University students	Yes	Frequency of ED use in the 6 months prior to the survey: rarely (1–10), sometimes (11–30), often (31–90) or very often (≥ 91 times). It also considers dual use with alcohol in the last 3 months	Self-administered questionnaire in the classroom
Martins A	2018	Portugal	School students	Yes	Frequency of ED consumption: daily, weekly, monthly or rarely; and, in a typical drinking day, number of units of ED drunk: 1, 2, 3; > 3	Self-administered questionnaire in the classroom
Scuri S	2018	Italy	School students	Yes	N/A	Self-administered questionnaire (no further description)
Thomas F	2018	UK	School students	No	Frequency of use of a series of food categories including ED	Self-administered online questionnaire
Totaro M	2018	Italy	School students	No	Weekly consumption of generic beverages and ED (alone or mixed with alcohol) in different times: during sport, parties and at home	Self-administered questionnaire in the classroom
Galimov A	2019	Germany	School students	Yes	Ever use of ED (e.g. Red Bull, Monster) and at least once a month	Face-to-face interviews by trained interviewers or supervised by teachers
Gruzieva TS	2019	Ukraine	University students	No	Weekly use of ED	Self-administered questionnaire (no further description)
Buja A	2020	Italy	School students	No	ED consumption: only rarely, on special occasions; some weekdays (Monday to Friday); only at the week-end; some weekdays (Monday to Friday) and at the weekend; and every day of the week	Self-administered questionnaire (computer-assisted)
Benkert R	2020	Switzerland	Adults (military)	Yes	Frequency of ED consumption more than once a day in the last 12 months	Self-administered questionnaire (printed form)
Cruz Munoz V	2020	Spain	School students	Yes	Consumption 1–2 times a month of each type of beverage in any given month of the last year	Self-administered questionnaire supervised by teachers in the classroom
Holguín EP	2020	Spain	University students	Yes	Ever use and monthly use of ED. It also considers occasional and frequent dual use of alcohol	Self-administered questionnaire (no further description)
Lebacq T	2020	Belgium	School students	Yes	More than once a week ED consumption (mentioned as ‘Energy drinks [Red bull®…]’)	Self-administered questionnaire in the classroom
Lehmann F	2020	Germany	School students	No	ED intake in the previous 4 weeks, including the quantity of drinks that the participants consumed	Face-to-face interviews by trained interviewers
Sljivo A	2020	Bosnia	University students	Yes	‘High chronic’ consumers were defined as consuming ED 4–5 times/week or more, and ‘high acute’ consumers as consuming at least 1 L/single session	Self-administered questionnaire (via social media)
Tanner T	2020	Finland	Adults (military)	No	Frequency of snack consumption, including ED every day or almost every day consumption	Self-administered questionnaire (computer-assisted)
Toth A	2020	Hungary	School and university students	Yes	Frequency of ED consumption: 1–2 small cans a month; 1–2 small cans a week; more than 2 small cans a week; 1 small can a day; and more than one small can a day. It also considers dual use with alcohol or with other caffeinated drinks	Self-administered questionnaire (printed form)
Błaszczyk-Bębenek E	2021	Poland	School students	No	Frequency and amount of caffeine-containing products: less frequently than once a week, 1–2 times a week, 3–4 times a week, 5–6 times a week, once a day, 2 times a day, 3 times a day	Self-administered questionnaire (no further description)
Boleslawska I	2021	Poland	Adults	No	Frequency of consumption of 26 products and 7 beverages: 1–3 times a month, once a week, several times a week, once a day, several times a day	Self-administered questionnaire (via various social media and email)
Brumboiu I	2021	France and Romania	University students	No	Consumption of soft enhancers, including ED, either sometimes or regularly, in the last 12 months	Self-administered online questionnaire
Dąbrowska-Galas M	2021	Poland	University students	No	Frequency of ED consumption: several times a month and several times a week	Self-administered questionnaire distributed by the researcher in the classroom
Fernandes S	2021	Portugal	Adults	No	Participants’ alcohol, stimulant drinks, illegal substances and pharmaceuticals consumption habits during the COVID-19 pandemic	Self-administered questionnaire (via various social media and email)
Franke AG	2021	Germany	University students and adults	No	Frequency of use of substances including ED as pharmacological neuroenhancement: during last month, during last year, longer than 1 year ago	Self-administered questionnaire via email
Halldorsson TI	2021	Iceland	School students	No	Frequency of ED consumption: more than once a week	Self-administered questionnaire in the classroom
Jebrini T	2021	Germany	University students	No	Frequency of ED consumption: once per year; once a month, once a week or more frequently	Self-administered online questionnaire
Kaldenbach S	2021	Norway	School students	Yes	Frequency of ED (Red Bull, Battery, etc.) consumption, ranging from ‘never’ to ‘several times a day’: ED consumers (ED <once a week or more) and high ED consumers (ED ≥ 4 times a week)	Self-administered online questionnaire supervised by teachers in the classroom
Morgan K	2021	UK	School students	No	Frequency of ED (such as Red Bull, Monster and Rockstar) consumption: weekly; daily or more	Self-administered questionnaire (no further description)
Oliver Angles A	2021	Spain	School students	Yes	Frequency of ED (Red bull®, Monster®, etc.) consumption in the last 7 d: daily, weekly	Self-administered questionnaire (no further description)
Puupponen M	2021	Finland	School students	Yes	Frequency of ED consumption: weekly, less than weekly	Self-administered questionnaire (printed form and via Webropol software)
Scalese M	2021	Italy	School students	Yes	Frequency of ED use (alone or with alcohol), in the lifetime, last year and last month: ‘never, once or twice, 3–9 times, 10–19 times and 20 times or more’. Respondents were considered current frequent users if they indicated that they had consumed ED on 20 or more occasions in the previous 30 d	Self-administered questionnaire (printed form or computer-based) in the classroom
Schroder H	2021	Spain	School students	Yes	Frequency of RSD, ED or sd consumption during the last year: less than 2 times/month, 1–5 times a week and more than 5 times a week	Self-administered questionnaire supervised by teachers in the classroom
Svensson A	2021	Sweden	School students	Yes	Ever use of ED (e.g. Red Bull, Monster, Burn, Power King, and other similar beverages) use and more than once a week use	Self-administered questionnaire via email
Vasic J	2021	Serbia	School students (refugee population)	No	Frequency of substance and alcohol use or abuse (including ED)	Self-administered questionnaire (no further description)
Atienza B	2022	Spain	University students	No	Lifetime and last 30 d’ prevalence of tobacco, alcohol, and several substances (including ED)	Self-administered online questionnaire
Brunborg GS	2022	Norway	School students	Yes	Frequency of ED consumption in the last 30 d: 1 d/month; 2–3 d/month; 1–2 d/week; 3–4 d week; every day or almost every day	Self-administered online questionnaire
Jeannou B	2022	France	Adults (athletes)	No	Habits of substance consumption (including ED)	Self-administered questionnaire (online or printed form)
Kaldenbach S	2022	Norway	School students	Yes	Frequency of ED (Red Bull, Battery, etc.) consumption: seldom; once a week; 2–3 times a week; more than 4 times a week	Self-administered online questionnaire
Kosendiak AA	2022	Poland	University students	No	Frequency of ED consumption: 1–3 times per month; once per week; few times per week; once per day; few times per day	Self-administered online questionnaire
Riera-Sampol A	2022	Spain	University students	No	Daily and weekly ED consumption	Self-administered online questionnaire
Sammito S	2022	Germany	Adults (military)	Yes	Frequency of use of dietary supplements such as ED	Self-administered online questionnaire
Soukiasian PD	2022	Greece	Adults	No	Frequency of use of dietary supplements such as ED	Face-to-face interviews by trained interviewers.
Tomanic M	2022	Serbia	School students	Yes	Frequency of ED use: weekly; every day; a few times daily	Self-administered questionnaire (printed form) in the classroom
Erdös A	2023	Hungary	University students	No	Frequency of ED consumption: weekly or less; several times a week; one portion per day; two portions per day; three portions per day; four portions per day; five or more portions per day	Self-administered online questionnaire
Kaldenbach S	2023	Norway	University students	Yes	Frequency of ED consumption: daily; 4–6 times per week; 2–3 times per week; 1 time per week; 1–3 times per month; seldom	Self-administered online questionnaire.
Mititelu M	2023	Romania	Adults	No	Frequency of ED consumption: daily; 2–3 times a week; once a week; 2–3 times a month; very rarely	Self-administered questionnaire (via various social media and email).
Pavlovic N	2023	Croacia	University students	Yes	Frequency of ED consumption, number of ED drank monthly, the type of most often consumed ED and level of ED consumption in one occasion	Self-administered online questionnaire
Petrauskiene S	2023	Lithuania	School students	No	Frequency of ED consumption: less than once a week; once a week; 2–4 d a week; 5–6 d a week; once a day; more than once a day	Self-administered questionnaire (no further description)
Protano C	2023	Italy	University students	Yes	Previous 6 months ED use: once a month; once a week; sometimes a week; once a day; more than once a day	Self-administered online questionnaire
Vogel C	2023	United Kingdom	School students	Yes	Daily ED consumption	Face-to-face interviews by researcher
Zivojinovic JI	2023	Serbia	University students	No	N/A	Self-administered questionnaire (printed form) in the classroom
Carrasco-Garrido P	2024	Spain	School students	No	Past 12 months ED consumption	Self-administered questionnaire (printed form) in the classroom
Di Martino G	2024	Italy	University students	No	Frequency of ED consumption before, during and after the COVID-19 lockdown: less than once a week; 1–3 times a week; more than 3 times a week	Self-administered online questionnaire
Kostecka J	2024	Poland	School students	No	Frequency of beverage consumption including ED	Self-administered questionnaire (printed form) in the classroom
Szepe O	2024	Hungary	University students	No	Frequency of ED consumption: every day; 2–4 times a week; occasionally	Self-administered online questionnaire
Vejrup K	2024	Norway	Adults (military)	No	Use of beverages including ED: 1–2 d; 3 or more days	Self-administered online questionnaire

The majority of studies (86 of 94) employed self-administered questionnaires as the primary method for data collection. Sixteen studies out of the eighty-six lacked any description of the setting in which the questionnaire was completed or the format of the questionnaire itself. The classroom emerged as the most frequent setting (*n* 27), with some studies (*n* 13) noting the involvement of teachers or research assistants in data collection. The most frequent format was self-administered online (*n* 28), followed by printed (*n* 13) and email formats (*n* 5). Seven studies collected data via face-to-face interviews (Table 3).

### Prevalence of energy drink consumption

Forty-nine of ninety-four studies aimed to estimate the prevalence of ED consumption as their primary objective (Table 3).

Supplemental Table 4 shows the prevalence of ED consumption by study year, population group and European country. Table 4 shows the prevalence of ED consumption in each study, grouped by population and based on the frequency of ED consumption. Fifty-one studies estimated the prevalence among school students, twenty-eight in university students and one in both groups^(51)^. The remaining eighteen studies assessed the prevalence of ED consumption in the general population, encompassing children, adolescents and/or adults.

**Table 4.** Prevalence of energy drink (ED) consumption in included studies (*n* 94) by population group

Author, year	Country	Prevalence of energy drink consumption																							
		Daily			Weekly			Monthly			Yearly			Ever			Regular			Occasional/infrequent			Mixed with alcohol		
		G	M	F	G	M	F	G	M	F	G	M	F	G	M	F	G	M	F	G	M	F	G	M	F
School students																									
Gambon DL, 2011	The Netherlands	**–**	–	–	**39·4**	45·7	32·3	**–**	–	–	**–**	–	–	**–**	–	–	**–**	–	–	**–**	–	–	**–**	–	–
James JE, 2011	Iceland	**38·0**	–	–	**–**	–	–	**–**	–	–	**–**	–	–	**–**	–	–	**–**	–	–	**–**	–	–	**–**	–	–
Gallimberti L, 2013	Italy	**1·3**	–	–	**5·5**	–	–	**6·5**	–	–	**–**	–	–	**33·3**	–	–	**–**	–	–	**19·9**	–	–	**–**	–	–
Kristjansson AL, 2013	Iceland	**20·2**	–	–	**–**	–	–	**–**	–	–	**–**	–	–	**–**	–	–	**–**	–	–	**–**	–	–	**–**	–	–
Flotta A, 2014	Italy	**–**	–	–	**–**	–	–	**–**	–	–	**–**	–	–	**67·7**	82·9	56·1	**–**	–	–	**–**	–	–	**46·1**	62·0	34·3
Gornicka M, 2014	Poland	**–**	–	–	**–**	–	–	**–**	–	–	**–**	–	–	**–**	–	–	**–**	–	–	**–**	–	–	**–**	–	–
Kristjansson AL, 2014^§^	Iceland	**–**	19·1	9·2	**–**	–	–	**–**	–	–	**–**	–	–	**–**	–	–	**–**	–	–	**–**	–	–	**–**	–	–
Vilija M, 2014	Lithuania	**21·0**	–	–	**–**	–	–	**–**	–	–	**–**	–	–	**–**	–	–	**–**	–	–	**–**	–	–	**–**	–	–
Gallimberti L, 2015^†,||^	Italy	**–**	–	–	**–**	–	–	**–**	18·7	7·4	**–**	–	–	**–**	45·0	24·8	**–**	–	–	**–**	–	–	**–**	5·4	1·4
Kristjansson AL, 2015^§^	Iceland	**–**	27·2	14·0	**–**	–	–	**–**	–	–	**–**	–	–	**–**	–	–	**–**	–	–	**–**	–	–	**–**	35·1	39·8
Liakoni E, 2015	Switzerland	**–**	–	–	**–**	–	–	**24·6**	–	–	**28·4**	–	–	**75·4**	–	–	**–**	–	–	**–**	–	–	**–**	–	–
Nowak D, 2015	Poland	**2·1**	3·2	1·2	**6·1**	8·1	4·4	**20·0**	20·1	19·9	**14·3**	11·9	16·1	**66·8**	74·6	60·8	**–**	–	–	**–**	–	–	**24·2**	10·6	13·6
Richards G, 2015^†^	United Kingdom	**8·6**	–	–	**16·0**	–	–	**30·6**	–	–	**–**	–	–	**–**	–	–	**–**	–	–	**–**	–	–	**–**	–	–
Barrense-Dias Y, 2016	Switzerland	**–**	–	–	**–**	–	–	**–**	–	–	**–**	–	–	**–**	–	–	**28·3**	36·3	20·1	**29·5**	–	–	**–**	–	–
Koivusilta L, 2016	Finland	**2·8**	4·2	1·5	**32·0**	38·7	25·7	**–**	–	–	**–**	–	–	**–**	–	–	**–**	–	–	**–**	–	–	**–**	–	–
Milavanovic DD, 2016	Serbia	**1·0**	–	–	**5·2**	–	–	**–**	–	–	**–**	–	–	**–**	–	–	**–**	–	–	**–**	–	–	**–**	–	–
Wierzejska R, 2016	Poland	**–**	–	–	**4·0**	–	–	**19·8**	–	–	**–**	–	–	**23·8**	–	–	**–**	–	–	**–**	–	–	**–**	–	–
Holubcikova J, 2017	Slovakia	**3·7**	–	–	**36·7**	–	–	**–**	–	–	**–**	–	–	**–**	–	–	**–**	–	–	**–**	–	–	**3·0**	–	–
Holubcikova J, 2017	Slovakia	**–**	–	–	**–**	–	–	**–**	–	–	**–**	–	–	**–**	–	–	**20·6**	27·6	13·6	**–**	–	–	**–**	–	–
Husarova D, 2017	Slovakia	**19·6**	–	–	**–**	–	–	**–**	–	–	**–**	–	–	**–**	–	–	**–**	–	–	**–**	–	–	**–**	–	–
Scalese M, 2017	Italy	**–**	–	–	**–**	–	–	**–**	–	–	**41·4**	53·9	28·7	**–**	–	–	**–**	–	–	**–**	–	–	**–**	–	–
Buja A, 2018	Italy	**–**	–	–	**–**	–	–	**14·7**	–	–	**–**	–	–	**–**	–	–	**–**	–	–	**–**	–	–	**–**	–	–
Degirmenci N, 2018	Norway	**–**	–	–	**52·3**	64·5	40·7	**–**	–	–	**–**	–	–	**–**	–	–	**–**	–	–	**–**	–	–	**–**	–	–
Martins A, 2018	Portugal	**–**	–	–	**14·1**	–	–	**34·0**	39·1	28·8	**–**	–	–	**56·7**	–	–	**–**	–	–	**–**	–	–	**–**	–	–
Scuri S, 2018	Italy	**–**	–	–	**–**	–	–	**–**	–	–	**–**	–	–	**–**	–	–	**–**	–	–	**–**	–	–	**–**	–	–
Thomas F, 2018	United Kingdom	**–**	–	–	**–**	–	–	**–**	–	–	**–**	–	–	**–**	–	–	**–**	–	–	**–**	–	–	**–**	–	–
Totaro M, 2018	Italy	**–**	–	–	**12·0**	–	–	**–**	–	–	**–**	–	–	**–**	–	–	**12·0**	–	–	**48·0**	–	–	**25·0**	–	–
Galimov A, 2019	Germany	**–**	–	–	**–**	–	–	**21·4**	28·3	14·1	**–**	–	–	**61·7**	68·4	54·9	**–**	–	–	**–**	–	–	**–**	–	–
Bruja A, 2020	Italy	**–**	–	–	**–**	–	–	**–**	–	–	**–**	–	–	**–**	–	–	**16·2**	–	–	**33·0**	–	–	**–**	–	–
Cruz Munoz V, 2020	Spain	**–**	–	–	**–**	–	–	**49·2**	–	–	**–**	–	–	**–**	–	–	**–**	–	–	**–**	–	–	**–**	–	–
Lebacq T, 2020	Belgium	**–**	–	–	**10·7**	14·9	8·1	**–**	–	–	**–**	–	–	**–**	–	–	**–**	–	–	**–**	–	–	**–**	–	–
Lehmann F, 2020	Germany	**–**	–	–	**–**	–	–	**8·9**	8·7	9·1	**–**	–	–	**–**	–	–	**–**	–	–	**–**	–	–	**–**	–	–
Toth A, 2020	Hungry	**6·4**	8·8	4·3	**12·4**	14·8	10·3	**–**	–	–	**–**	–	–	**31·0**	–	–	**–**	–	–	**–**	–	–	**7·4**	–	–
Vasic J, 2021	Bosnia	**–**	–	–	**–**	–	–	**–**	–	–	**–**	–	–	**–**	–	–	**–**	–	–	**–**	–	–	**–**	–	–
Błaszczyk-Bębenek E, 2021	Poland	**4·7**	–	–	**22·2**	–	–	**–**	–	–	**–**	–	–	**–**	–	–	**–**	–	–	**–**	–	–	**–**	–	–
Halldorsson TI, 2021	Iceland	**–**	–	–	**37·2**	38·4	36·0	**–**	–	–	**–**	–	–	**–**	–	–	**–**	–	–	**–**	–	–	**–**	–	–
Kaldenbach S, 2021^†^	Norway	**–**	–	–	**–**	–	–	**–**	–	–	**54·4**	65·7	43·6	**–**	–	–	**–**	–	–	**–**	–	–	**–**	–	–
Morgan K, 2021	UK	**6·0**	–	–	**17·0**	–	–	**–**	–	–	**–**	–	–	**–**	–	–	**–**	–	–	**–**	–	–	**–**	–	–
Oliver Angles A, 2021	Spain	**11·9**	16·9	7·1	**30·9**	43·6	18·7	**–**	–	–	**–**	–	–	**–**	–	–	**–**	–	–	**–**	–	–	**–**	–	–
Puupponen M, 2021^†^	Finland	**–**	–	–	**24·4**	32·1	16·8	**–**	–	–	**–**	–	–	**50·4**	59·6	41·1	**–**	–	–	**–**	–	–	**–**	–	–
Scalese M, 2021^†^	Italy	**–**	–	–	**–**	–	–	**–**	45·4	20·3	**–**	63·4	38·3	**–**	75·7	61·8	**–**	4·3	0·8	**–**	–	–	**–**	19·4^‡^	9·6^‡^
Schroder H, 2021	Spain	**1·3**	1·7	1·0	**11·0**	15·7	6·9	**–**	–	–	**–**	–	–	**–**	–	–	**–**	–	–	**–**	–	–	**–**	–	–
Svensson A, 2021^†^	Sweden	**2·3**	4·7	2·6	**13·0**	18·1	8·6	**–**	–	–	**–**	–	–	**–**	–	–	**–**	–	–	**–**	–	–	**–**	–	–
Brunborg GS, 2022	Norway	**1·0**	–	–	**9·0**	–	–	**28·0**	–	–	**–**	–	–	**–**	–	–	**–**	–	–	**–**	–	–	**–**	–	–
Kaldenbach S, 2022	Norway	**–**	–	–	**18·9**	–	–	**–**	–	–	**–**	–	–	**–**	–	–	**–**	–	–	**32·0**	–	–	**–**	–	–
Tomanic M, 2022	Serbia	**–**	–	–	**17·1**	19·8	15·6	**–**	–	–	**–**	–	–	**–**	–	–	**–**	–	–	**–**	–	–	**–**	–	–
Petrauskiene S, 2023	Lithuania	**–**	–	–	**–**	–	–	**–**	–	–	**–**	–	–	**34·8**	–	–	**–**	–	–	**–**	–	–	**–**	–	–
Vogel C, 2023	United Kingdom	**7·0**	7·8	6·3	**–**	–	–	**–**	–	–	**–**	–	–	**–**	–	–	**–**	–	–	**–**	–	–	**–**	–	–
Carrasco-Garrido P, 2024^†^	Spain	**–**	–	–	**–**	–	–	**–**	–	–	**39·9**	49·2	30·9	**–**	–	–	**–**	–	–	**–**	–	–	**–**	–	–
Kostecka J, 2024	Poland	**–**	–	–	**–**	–	–	**–**	–	–	**–**	–	–	**–**	–	–	**–**	–	–	**–**	–	–	**–**	–	–
**University students**																									
Oteri A, 2007	Italy	**–**	–	–	**–**	–	–	**–**	–	14·8	**–**	–	–	**–**	–	–	**–**	–	–	**–**	–	–	**39·8**	–	–
Maier LJ, 2013	Switzerland	**4·0**	–	–	**–**	–	–	**29·7**	–	–	**–**	–	–	**67·5**	–	–	**–**	–	–	**–**	–	–	**–**	–	–
Ravelo Abreu A, 2013	Spain	**31·4 (exam periods)**	–	–	**11·4**	–	–	**–**	–	–	**–**	–	–	**82·7**	–	–	**–**	–	–	**–**	–	–	**30·3**	–	–
Gornicka M, 2014	Poland	**–**	–	–	**–**	–	–	**–**	–	–	**–**	–	–	**–**	–	–	**–**	–	–	**–**	–	–	**–**	–	–
Chuda A, 2014	Poland	**1·5**	4·7	0·0	**3·1**	2·3	3·4	**21·4**	18·6	22·7	**–**	–	–	**–**	–	–	**–**	–	–	**42·0**	60·5	33·0	**–**	–	–
Casuccio A, 2015	Italy	**4·0**	–	–	**32·0**	–	–	**30·0**	–	–	**–**	–	–	**–**	–	–	**22·0**	60·0	40·0	**34·0**	–	–	**49·0**	–	–
Cencek P, 2016	Poland	**61·8**	–	–	**–**	–	–	**–**	–	–	**–**	–	–	**78·5**	–	–	**–**	–	–	**–**	–	–	**–**	–	–
Parezanovic GS, 2016	Serbia	**1·6**	–	–	**11·2**	32·0	–	**–**	–	–	**–**	–	–	**–**	–	–	**–**	–	–	**–**	–	–	**–**	–	–
Vitiello v, 2016	Italy	**–**	–	–	**64·5**	–	–	**–**	–	–	**–**	–	–	**75·8**	78·9	57·4	**–**	–	–	**–**	–	–	**72·1**	62·8	37·2
Concerto C, 2017	Italy	**–**	–	–	**48·6**	–	–	**–**	–	–	**–**	–	–	**–**	–	–	**–**	–	–	**–**	–	–	**–**	–	–
Majori S, 2018	Italy	**–**	–	–	**–**	–	–	**–**	–	–	**–**	–	–	**–**	–	–	**–**	–	–	**–**	–	–	**25·4**	38·6	19·7
Gruzieva TS, 2019	Ukraine	**–**	–	–	**32·5**	–	–	**–**	–	–	**–**	–	–	**–**	–	–	**–**	–	–	**–**	–	–	**13·6**	–	–
Holguín EP, 2020	Spain	**–**	–	–	**–**	–	–	**32·6**	53·8	23·5	**–**	–	–	**83·9**	–	–	**–**	–	–	**–**	–	–	**17·6**	–	–
Sljivo A, 2020	Bosnia	**–**	–	–	**–**	–	–	**–**	–	–	**61·7**	67·3	59·7	**–**	–	–	**–**	–	–	**–**	–	–	**13·2**	–	–
Toth A, 2020	Hungry	**6·4**	8·8	4·3	**12·4**	14·8	10·3	**–**	–	–	**–**	–	–	**31·0**	–	–	**–**	–	–	**–**	–	–	**7·4**	–	–
Brumboiu I, 2021	France	**–**	–	–	**–**	–	–	**–**	–	–	**8·2**	–	–	**–**	–	–	**–**	–	–	**–**	–	–	**–**	–	–
Brumboiu I, 2021	Romania	**–**	–	–	**–**	–	–	**–**	–	–	**17·4**	–	–	**–**	–	–	**–**	–	–	**–**	–	–	**–**	–	–
Dąbrowska-Galas M, 2021	Poland	**–**	–	–	**7·5**	–	–	**9·8**	–	–	**–**	–	–	**–**	–	–	**–**	–	–	**–**	–	–	**–**	–	–
Jebrini T, 2021^§^	Germany	**–**	–	–	**12·7**	–	–	**11·0**	–	–	**22·0**	–	–	**45·7**	–	–	**–**	–	–	**–**	–	–	**–**	–	–
Atienza B, 2022	Spain	**–**	–	–	**–**	–	–	**13·0**	–	–	**–**	–	–	**50·0**	–	–	**–**	–	–	**–**	–	–	**–**	–	–
Kosendiak AA, 2022^†^	Poland	**0·5**	–	–	**15·5**	–	–	**29·0**	–	–	**–**	–	–	**–**	–	–	**–**	–	–	**–**	–	–	**–**	–	–
Riera-Sampol A, 2022	Spain	**10·7**	18·0	7·4	**–**	–	–	**–**	–	–	**–**	–	–	**–**	–	–	**–**	–	–	**–**	–	–	**–**	–	–
Erdös A, 2023	Hungary	**4·7**	–	–	**20·9**	–	–	**–**	–	–	**–**	–	–	**70·3**	–	–	**–**	–	–	**–**	–	–	**–**	–	–
Kaldenbach S, 2023	Norway	**–**	4·7	3·3	**–**	35·4	27·9	**–**	20·2	19·2	**–**	–	–	**–**	–	–	**–**	–	–	**–**	–	–	**–**	–	–
Pavlovic N, 2023	Croatia	**–**	–	–	**12·6**	–	–	**52·4**	64·0	48·2	**–**	–	–	**–**	–	–	**–**	–	–	**–**	–	–	**39·4**	48·6	36·1
Protano C, 2023	Italy	**2·1**	–	–	**6·8**	–	–	**6·3**	–	–	**–**	–	–	**15·2**	–	–	**–**	–	–	**–**	–	–	**–**	–	–
Zivojinovic JI, 2023	Serbia	**–**	–	–	**–**	–	–	**–**	–	–	**–**	–	–	**–**	–	–	**–**	–	–	**–**	–	–	**–**	–	–
Di Martino G, 2024	Italy	**–**	–	–	**9·9**	–	–	**18·1**	–	–	**–**	–	–	**–**	–	–	**–**	–	–	**–**	–	–	**–**	–	–
Szepe O, 2024	Hungary	**2·7**	–	–	**8·6**	–	–	**–**	–	–	**–**	–	–	**–**	–	–	**–**	–	–	**22·5**	–	–	**–**	–	–
**General population**																									
**Children**																									
Zucconi S, 2013	16 European countries	**–**	–	–	**–**	–	–	**–**	–	–	**18·0**	22·0	14·0	**–**	–	–	**–**	–	–	**–**	–	–	**–**	–	–
Zucconi S, 2013	United Kingdom	**–**	–	–	**–**	–	–	**–**	–	–	**24·0**	–	–	**–**	–	–	**–**	–	–	**–**	–	–	**–**	–	–
Zucconi S, 2013	Sweden	**–**	–	–	**–**	–	–	**–**	–	–	**14·0**	–	–	**–**	–	–	**–**	–	–	**–**	–	–	**–**	–	–
Zucconi S, 2013	Spain	**–**	–	–	**–**	–	–	**–**	–	–	**26·0**	–	–	**–**	–	–	**–**	–	–	**–**	–	–	**–**	–	–
Zucconi S, 2013	Romania	**–**	–	–	**–**	–	–	**–**	–	–	**12·0**	–	–	**–**	–	–	**–**	–	–	**–**	–	–	**–**	–	–
Zucconi S, 2013	Poland	**–**	–	–	**–**	–	–	**–**	–	–	**12·0**	–	–	**–**	–	–	**–**	–	–	**–**	–	–	**–**	–	–
Zucconi S, 2013	Netherlands	**–**	–	–	**–**	–	–	**–**	–	–	**20·0**	–	–	**–**	–	–	**–**	–	–	**–**	–	–	**–**	–	–
Zucconi S, 2013	Italy	**–**	–	–	**–**	–	–	**–**	–	–	**17·0**	–	–	**–**	–	–	**–**	–	–	**–**	–	–	**–**	–	–
Zucconi S, 2013	Hungary	**–**	–	–	**–**	–	–	**–**	–	–	**6·0**	–	–	**–**	–	–	**–**	–	–	**–**	–	–	**–**	–	–
Zucconi S, 2013	Greece	**–**	–	–	**–**	–	–	**–**	–	–	**10·0**	–	–	**–**	–	–	**–**	–	–	**–**	–	–	**–**	–	–
Zucconi S, 2013	Germany	**–**	–	–	**–**	–	–	**–**	–	–	**13·0**	–	–	**–**	–	–	**–**	–	–	**–**	–	–	**–**	–	–
Zucconi S, 2013	France	**–**	–	–	**–**	–	–	**–**	–	–	**22·0**	–	–	**–**	–	–	**–**	–	–	**–**	–	–	**–**	–	–
Zucconi S, 2013	Finland	**–**	–	–	**–**	–	–	**–**	–	–	**18·0**	–	–	**–**	–	–	**–**	–	–	**–**	–	–	**–**	–	–
Zucconi S, 2013	Czech Republic	**–**	–	–	**–**	–	–	**–**	–	–	**40·0**	–	–	**–**	–	–	**–**	–	–	**–**	–	–	**–**	–	–
Zucconi S, 2013	Cyprus	**–**	–	–	**–**	–	–	**–**	–	–	**11·0**	–	–	**–**	–	–	**–**	–	–	**–**	–	–	**–**	–	–
Zucconi S, 2013	Belgium	**–**	–	–	**–**	–	–	**–**	–	–	**8·0**	–	–	**–**	–	–	**–**	–	–	**–**	–	–	**–**	–	–
Zucconi S, 2013	Austria	**–**	–	–	**–**	–	–	**–**	–	–	**9·0**	–	–	**–**	–	–	**–**	–	–	**–**	–	–	**–**	–	–
**Adolescents**																									
Zucconi S, 2013	16 European countries	**–**	28·0	–	**–**	–	–	**–**	–	–	**68·0**	74·0	63·0	**–**	–	–	**–**	–	–	**–**	–	–	**–**	–	–
Zucconi S, 2013	United Kingdom	**–**	–	–	**–**	–	–	**–**	–	–	**69·0**	–	–	**–**	–	–	**–**	–	–	**–**	–	–	**–**	–	–
Zucconi S, 2013	Sweden	**–**	–	–	**–**	–	–	**–**	–	–	**69·0**	–	–	**–**	–	–	**–**	–	–	**–**	–	–	**–**	–	–
Zucconi S, 2013	Spain	**–**	–	–	**–**	–	–	**–**	–	–	**62·0**	–	–	**–**	–	–	**–**	–	–	**–**	–	–	**–**	–	–
Zucconi S, 2013	Romania	**–**	–	–	**–**	–	–	**–**	–	–	**70·0**	–	–	**–**	–	–	**–**	–	–	**–**	–	–	**–**	–	–
Zucconi S, 2013	Poland	**–**	–	–	**–**	–	–	**–**	–	–	**73·0**	–	–	**–**	–	–	**–**	–	–	**–**	–	–	**–**	–	–
Zucconi S, 2013	Netherlands	**–**	–	–	**–**	–	–	**–**	–	–	**67·0**	–	–	**–**	–	–	**–**	–	–	**–**	–	–	**–**	–	–
Zucconi S, 2013	Italy	**–**	–	–	**–**	–	–	**–**	–	–	**56·0**	–	–	**–**	–	–	**–**	–	–	**–**	–	–	**–**	–	–
Zucconi S, 2013	Hungary	**–**	–	–	**–**	–	–	**–**	–	–	**78·0**	–	–	**–**	–	–	**–**	–	–	**–**	–	–	**–**	–	–
Zucconi S, 2013	Greece	**–**	–	–	**–**	–	–	**–**	–	–	**48·0**	–	–	**–**	–	–	**–**	–	–	**–**	–	–	**–**	–	–
Zucconi S, 2013	Germany	**–**	–	–	**–**	–	–	**–**	–	–	**60·0**	–	–	**–**	–	–	**–**	–	–	**–**	–	–	**–**	–	–
Zucconi S, 2013	France	**–**	–	–	**–**	–	–	**–**	–	–	**66·0**	–	–	**–**	–	–	**–**	–	–	**–**	–	–	**–**	–	–
Zucconi S, 2013	Finland	**–**	–	–	**–**	–	–	**–**	–	–	**65·0**	–	–	**–**	–	–	**–**	–	–	**–**	–	–	**–**	–	–
Zucconi S, 2013	Czech Republic	**–**	–	–	**–**	–	–	**–**	–	–	**82·0**	–	–	**–**	–	–	**–**	–	–	**–**	–	–	–	–	–
Zucconi S, 2013	Cyprus	**–**	–	–	**–**	–	–	**–**	–	–	**53·0**	–	–	**–**	–	–	**–**	–	–	**–**	–	–	–	–	–
Zucconi S, 2013	Belgium	**–**	–	–	**–**	–	–	**–**	–	–	**85·0**	–	–	**–**	–	–	**–**	–	–	**–**	–	–	–	–	–
Zucconi S, 2013	Austria	**–**	–	–	**–**	–	–	**–**	–	–	**75·0**	–	–	**–**	–	–	**–**	–	–	**–**	–	–	–	–	–
**Adults**																									
Braun H, 2009	Germany	**–**	–	–	**–**	–	–	**–**	–	–	**–**	–	–	–	–	–	–	–	–	**–**	–	–	**–**	–	–
Zucconi S, 2013	16 European countries	**–**	–	–	**–**	–	–	**–**	–	–	**30·0**	37·0	26·0	–	–	–	–	–	–	**–**	–	–	**17·0**	21·0	14·0
Zucconi S, 2013	United Kingdom	**–**	–	–	**–**	–	–	**–**	–	–	**28·0**	–	–	–	–	–	–	–	–	**–**	–	–	**16·0**	–	–
Zucconi S, 2013	Sweden	**–**	–	–	**–**	–	–	**–**	–	–	**22·0**	–	–	–	–	–	–	–	–	**–**	–	–	**11·0**	–	–
Zucconi S, 2013	Spain	**–**	–	–	**–**	–	–	**–**	–	–	**31·0**	–	–	–	–	–	–	–	–	**–**	–	–	**15·0**	–	–
Zucconi S, 2013	Romania	**–**	–	–	**–**	–	–	**–**	–	–	**38·0**	–	–	–	–	–	–	–	–	**–**	–	–	**25·0**	–	–
Zucconi S, 2013	Poland	**–**	–	–	**–**	–	–	**–**	–	–	**45·0**	–	–	–	–	–	–	–	–	**–**	–	–	**19·0**	–	–
Zucconi S, 2013	Netherlands	**–**	–	–	**–**	–	–	**–**	–	–	**21·0**	–	–	–	–	–	–	–	–	**–**	–	–	**10·0**	–	–
Zucconi S, 2013	Italy	**–**	–	–	**–**	–	–	**–**	–	–	**28·0**	–	–	–	–	–	–	–	–	**–**	–	–	**17·0**	–	–
Zucconi S, 2013	Hungary	**–**	–	–	**–**	–	–	**–**	–	–	**40·0**	–	–	–	–	–	–	–	–	**–**	–	–	**19·0**	–	–
Zucconi S, 2013	Greece	**–**	–	–	**–**	–	–	**–**	–	–	**32·0**	–	–	–	–	–	–	–	–	**–**	–	–	**17·0**	–	–
Zucconi S, 2013	Germany	**–**	–	–	**–**	–	–	**–**	–	–	**30·0**	–	–	–	–	–	–	–	–	**–**	–	–	**20·0**	–	–
Zucconi S, 2013	France	**–**	–	–	**–**	–	–	**–**	–	–	**22·0**	–	–	–	–	–	–	–	–	**–**	–	–	**12·0**	–	–
Zucconi S, 2013	Finland	**–**	–	–	**–**	–	–	**–**	–	–	**29·0**	–	–	–	–	–	–	–	–	**–**	–	–	**14·0**	–	–
Zucconi S, 2013	Czech Republic	**–**	–	–	**–**	–	–	**–**	–	–	**46·0**	–	–	–	–	–	–	–	–	**–**	–	–	**27·0**	–	–
Zucconi S, 2013	Cyprus	**–**	–	–	**–**	–	–	**–**	–	–	**14·0**	–	–	–	–	–	–	–	–	**–**	–	–	**3·0**	–	–
Zucconi S, 2013	Belgium	**–**	–	–	**–**	–	–	**–**	–	–	**15·0**	–	–	–	–	–	–	–	–	**–**	–	–	**8·0**	–	–
Zucconi S, 2013	Austria	**–**	–	–	**–**	–	–	**–**	–	–	**50·0**	–	–	**–**	–	–	**–**	–	–	**–**	–	–	**29·0**	–	–
Rudolph E, 2014	Austria	**–**	–	–	**–**	–	–	**–**	–	–	**–**	–	–	**–**	–	–	**–**	–	–	**–**	–	–	**–**	–	–
Friis K, 2014	Denmark	**–**	–	–	**–**	–	–	**–**	–	–	**–**	–	–	**–**	–	–	**–**	–	–	**–**	–	–	**–**	–	–
Wardenaar F, 2016	The Netherlands	**–**	–	–	**–**	–	–	**–**	–	–	**–**	–	–	**–**	–	–	**–**	–	–	**–**	–	–	**–**	–	–
Pacifici R, 2016	Italy	**–**	–	–	**–**	–	–	**–**	–	–	**–**	–	–	**–**	–	–	**–**	–	–	**–**	–	–	**–**	–	–
Treur JL, 2016	The Netherlands	**–**	–	–	**–**	5·9	4·4	**–**	–	–	**–**	–	–	**–**	5·8	4·4	**–**	–	–	**–**	–	–	**–**	–	–
Casuccio A, 2017	Italy	**–**	–	–	**49·1**	–	–	**50·9**	–	–	**–**	–	–	**–**	–	–	**77·9**	–	–	**–**	–	–	**72·0**	–	–
Benkert R, 2020^†,*^	Switzerland	**6·1**	–	–	**–**	–	–	**–**	–	–	**–**	–	–	**–**	–	–	**–**	–	–	**–**	–	–	**–**	–	–
Tanner T, 2020^*^	Finland	**–**	28·8	–	**–**	–	–	**–**	–	–	**–**	–	–	**–**	–	–	**–**	–	–	**–**	–	–	**–**	–	–
Boleslawska I, 2021^†^	Poland	**1·3**	–	–	**5·1**	–	–	**8·3**	–	–	**–**	–	–	**–**	–	–	**–**	–	–	**–**	–	–	**–**	–	–
Fernandes S, 2021	Portugal	**–**	–	–	**–**	–	–	**–**	–	–	**–**	–	–	**–**	–	–	**–**	–	–	**–**	–	–	**–**	–	–
Franke AG, 2021	Germany	**–**	–	–	**–**	–	–	**31·6**	–	–	**49·2**	–	–	**62·8**	–	–	**–**	–	–	**–**	–	–	**–**	–	–
Jeannou B, 2022^**^	France	**–**	–	–	**–**	–	–	**–**	–	–	**13·2**	–	–	**–**	–	–	**–**	–	–	**–**	–	–	**–**	–	–
Sammito S, 2022^*^	Germany	**–**	–	–	**16·0**	–	–	**–**	–	–	**–**	–	–	**–**	–	–	**–**	–	–	**–**	–	–	**–**	–	–
Soukiasian PD, 2022	Germany	**–**	–	–	**–**	–	–	**–**	–	–	**–**	–	–	**–**	–	–	**–**	–	–	**–**	–	–	**–**	–	–
Mititelu M, 2023	Romania	**1·8**	3·1	1·5	**7·5**	12·2	6·4	**4·6**	7·6	3·9	**–**	–	–	**–**	–	–	**–**	–	–	**–**	–	–	**–**	–	–
Vejrup K, 2024^*,¶^	Norway	**31·0**	–	–	**–**	–	–	**–**	–	–	**–**	–	–	**–**	–	–	**–**	–	–	**–**	–	–	**–**	–	–
Adults and adolescents^***^																									
Braun H, 2009^*^	Germany	**–**	–	–	**–**	–	–	**–**	–	25·0	**–**	–	–	**–**	–	–	**–**	–	–	**–**	–	–	**–**	–	–
Friis K, 2014	Denmark	**–**	–	–	**15·8**	23·9	7·1	**–**	–	–	**–**	–	–	**–**	–	–	**–**	–	–	**–**	–	–	**–**	–	–
Rudolph E, 2014	Austria	**–**	–	–	**–**	–	–	**–**	–	–	**–**	–	–	**–**	–	–	**62·0**	–	–	**–**	–	–	**–**	–	–
Pacifici R, 2016	Italy	**–**	–	–	**–**	–	–	**–**	–	–	**–**	–	–	**20·1**	28·8	11·4	**–**	–	–	**–**	–	–	**–**	–	–
Wardenaar F, 2016	The Netherlands	**–**	–	–	**–**	–	–	**–**	–	–	**22·0**	27·0	16·0	**–**	–	–	**–**	–	–	**–**	–	–	**–**	–	–

G: global; M: male; F: female.

The use of boldface is intended to highlight the overall prevalences, making key data points more visually distinct and thereby facilitating easier and quicker interpretation of the table.

† Prevalence from the most recent year.

‡ Prevalence from the last month.

§ Overall prevalence derived from the sum of the different intensity-based consumption percentages.

|| Overall prevalence derived from the sum of the different prevalences stratified by age group or academic year.

¶ Prevalence from field exercise setting (higher prevalence).

* Military population.

** Athletes.

*** This section includes studies carried out in the adult and adolescent population without differentiating between these two groups.

Fifty-four studies assessed global prevalence across different time frames. The most studied frequency was weekly (*n* 45), followed by daily (*n* 35) and monthly (*n* 28) (Table 4). The study by Nowak^(52)^ examined the frequency of consumption in detail, whereas seven studies did not specify the frequency^(8,16,26,48,53)^.

The global prevalence of daily consumption varied between 0·5 % in 2022^(54)^ and 61·8 % in 2016^(55)^, both among Polish university students. Global prevalence of weekly consumption ranged from 3·1 % among Polish university students in 2014^(49)^ to 64·5 % among Italian university students in 2016^(56)^. A single study, published in 2013^(7)^, estimated the prevalence of yearly ED consumption globally and across sixteen European countries, encompassing children, adolescents and adults; the prevalence by country ranged from 6·0 % among Hungarian children to 85·0 % among Belgian adolescents. With regard to studies that estimate lifetime consumption, global prevalence varied from 20·1 % among Italian adolescents and adults in 2016^(57)^ to 83·9 % among Spanish university students in 2020^(58)^. A total of thirty-seven studies, irrespective of the frequency of ED consumption, stratified the prevalence by participant’s sex. In all, higher consumption was observed among males compared to females (Table 4).

Nineteen studies ascertained the prevalence of dual consumption of ED and alcohol (Table 4). The prevalence ranged from 3 % among Slovakian students in 2017^(59)^ to 72·2 % among Italian adults in 2017^(60)^. Interestingly, out of the nine studies that stratified the prevalence of consumption by sex, two studies^(52,61)^ pointed out in 2015 that Icelandic and Polish school-aged girls consumed mixed ED and alcohol more frequently than boys (39·8 % *v*. 35·1 % and 13·6 % *v*. 10·6 %, respectively).

### Characterisation of energy drink consumers

Table 5 shows the studies that characterised ED consumers (*n* 23). Fourteen studies characterised the school-age population, with weekly (*n* 8) and lifetime (*n* 3) consumption being the predominant frequencies. Italy stands out as the country with the majority of these studies (*n* 5). Five studies accounted for potential differences in characterisation based on participant’s sex, age or study period^(30,33,44,62)^. All studies collectively considered seventy-five different variables, with participant’s sex, age, tobacco smoking, alcohol consumption, physical activity and socio-economic-related variables being the most frequently examined. The socio-economic-related variables included aspects such as socio-economic status, household income, family affluence and structure, ethnic and migrant background, parental educational level, maternal origin, paternal and maternal occupation, place of residence and educational level. Fifty-nine variables were found to be associated with ED consumption in at least one study. Male gender, the socio-economic-related variables, tobacco and alcohol consumption were the most frequently associated variables (Table 5).

**Table 5.**
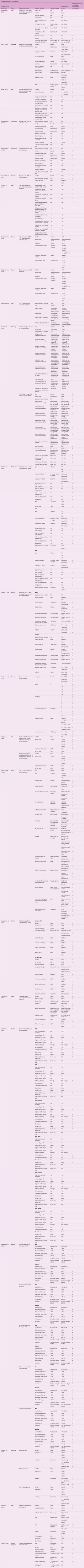
Characterisation of energy drink (ED) consumers (*n* 23)

Of those characteristics assessed in more than one study, it was agreed that low academic performance^(29,32,63)^, lower socio-economic status^(28,30,64)^, having sexual relationships^(29,65)^, having accidents^(29,66)^ and going out with friends^(29,66)^ were associated with greater ED consumption in students. Tranquilizers/anxiolytics were associated with greater ED consumption when valued both in students^(66)^ and the military^(44)^.

### Evaluation of the quality of the studies

When assessing quality, seventy-six studies were deemed to be of low quality, while the remaining eighteen were rated as moderate (see online supplementary material, Supplemental Table 3). The mean score was 6·43 and median score was 7. Several factors contributed to these ratings, including inadequate representation of the target population, lack of justification for sample size and low response rates. The lack of comprehensive specifications for self-administered questionnaires and inadequate supervision of interviewers also contributed to these ratings. It is noteworthy that a significant proportion of studies (*n* 70) only provided a descriptive analysis. Scores varied between 0 and 12, with Scuri et al.^(48)^ having the lowest score and both Zucconi et al.^(7)^ and Holubcikova et al.^(67)^ having the highest. Authors of the studies with lower scores reported lack of representativeness and differences in the method of administration of the questionnaire as the main methodological limitations; while studies with higher scores reported as limitations the cross-sectional design, the self-administration of the questionnaire and the possible influence on the results of assessing multiple comparisons.

## Discussion

This study represents the first review to analyse ED consumption prevalence across twenty-seven European countries over a 17-year period. In addition to providing global, sex-stratified and frequency-stratified prevalence data, the analysis specifically considers the definitions of consumption provided by the selected studies, all possible consumption patterns and the detailed characterisation of consumers. The most studied frequency was weekly consumption, and the most studied population was school students. In studies that tracked ED consumption over time, an increase in prevalence was observed. It was not possible to estimate an overall value for the prevalence of consumption due to the great variability in its assessment. Characteristics most related to consumption were low socio-economic status, alcohol and tobacco consumption, physical activity, age and sex.

### Prevalence of consumption

The assessment of temporal trends in ED consumption prevalence and differences in ED consumption by year and country was not possible due to the significant variability among studies. This diversity encompasses differences in study design, study populations, measurement methods and definitions of consumption. The populations under study have different demographic and socio-economic characteristics that condition their consumption. In turn, the measurement methods varied from brief questionnaires, through an in-depth battery of questions, face-to-face interviews to online surveys. The studies included have different approaches to consumption, and their definition relies on the establishment of variable time frames of evocation, from daily to lifetime, and a wide variability in the characterisation of frequency and/or intensity. Some studies did not even evoke recall to a specific time frame and used approximations of frequency and intensity based on imprecise aspects such as occasional or regular consumption. These differences prevent the comparison of ED prevalence estimations.

The eight studies that assessed trends in ED consumption identified an increasing trend, with the study by Scalese et al.^(31)^ being the one that covered the longest period, from 2008 to 2019. Data derived from independent studies developed in the same population are difficult to compare, mainly due to changes in the methodology or the definition of consumption.

In studies that use the same methodology to estimate prevalence in different population groups, prevalence varies. An example of this is the study by Zucconi et al.^(7)^, a study of the European population performed in 2012 that estimated the prevalence of ED consumption in children, adolescents and adults. The prevalence varied between these three groups, with adolescents having higher consumption. This study^(7)^ shows variations between countries. For example, the Czech Republic is one of the countries with the highest prevalence of consumption for all age groups, and Cyprus is one of the countries with the lowest prevalence. The individual studies identified endorse these results, with the eastern European countries showing higher prevalence. This is consistent with studies that relate poor health status in these populations to lack of health and behavioural information, greater belief in uncontrollable influences and lower emotional well-being, which are associated with unhealthy lifestyles^(68)^. In relation to Cyprus, there are no other published studies with prevalence estimations.

High variability of the prevalence of ED consumption was also found in another study performed by Aonso-Diego et al.^(69)^ about worldwide ED consumption. This study presented their meta-analysis results, even though they were not conclusive due to high heterogeneity.

The disparity in prevalence rates is such that even within the same population group, country and year, the prevalence differs significantly. For example, in 2012 in Italy, Gallimberti et al.^(70)^ estimated that 33·3 % of school students had ever consumed ED, while Flotta et al.^(65)^ estimated a prevalence of 67·7 % for the same frequency of consumption. To date, the impact of the wording of the questions ascertaining prevalence on ED consumption has not been evaluated. However, it is a crucial factor to consider, as subtle differences in how questions are phrased can potentially influence respondents’ answers and subsequently affect prevalence estimates.

High variability in the study results may be due to questionnaire characteristics and their administration, especially inadequate supervision of interviewers. Different methods to ascertain prevalence have different biases that could result in implications for the validity of the research, and for the evidence used for public policy making^(71)^. In addition, inadequate representation of the target population, lack of justification for sample size and low response rates may also have introduced selection and non-response bias.

In general, most European countries do not have a clear picture of the full scenario of the consumption in their population. As mentioned, most studies focused on school and university students. Furthermore, most countries have few studies conducted to assess ED consumption prevalence. These studies often do not follow the same methodology, so they are not useful for describing the evolution of consumption.

### Characteristics of consumers

Most of the studies that assessed sex identified that being male increased the probability of ED consumption. Many explanations were postulated to describe this association. The most widely accepted is that it is related to the marketing campaigns that associate ED with extreme sports and physical, sexual and academic performance aimed at men^(5,9–15)^. This is also an explanation used to associate higher ED consumption with enhanced physical activity. Prevalence in athletes should stand out due to marketing campaigns directed at them and the misinformation that these drinks are good for those who practice sports^(11–13)^. Once again, the supposed higher prevalence of consumption among athletes could not be verified due to a lack of consistency in methodologies.

Another possible explanation is the difference in the motive for consumption. According to Branco et al., females consume out of curiosity^(72)^. On the other hand, some studies link higher male consumption with higher screen time^(27,30,62,64)^ and gaming^(73)^. This is consistent with the results obtained by studies which associated ED consumption with marketing campaigns and social media, specifically Twitch^(74–76)^. In general, male sex is related to unhealthier lifestyles^(77)^. The higher ED consumption was also related to traditional masculinity ideology, hypermasculinity and jock identity^(14,78)^. It can be reasonably inferred that, regardless of which of the various postulated explanations are considered for the higher consumption of ED in men, they are all related to gender aspects that the industry has been able to exploit. The concept of masculinity has long been regarded as a potential risk factor for men’s health behaviours, given the assumption that it may encourage engagement in risky practices. Extreme sports, enhancement of physical and sexual performance and gaming are all practices historically associated with the concept of masculinity^(78)^. The industry, recognising this as an opportunity, targeted this group by leveraging gender-related issues. For example, brands designed specific packaging for these beverages exclusively for men^(62)^. In this way, their marketing campaigns became more focused on targeting the aforementioned characteristics.

It is worth noting that Kaldenbach et al.^(27)^ found that, in students, although consumption was still higher in males, ED use in females was increasing. Among those who found an association with sex, the studies that did not find greater consumption in males were anecdotal^(27,70)^.

Higher age was associated with greater consumption in school students^(29,63,67)^, while younger age was associated with greater consumption in university students^(9,16)^. Those studies that assessed the age of first consumption estimated that most people begin before age 13^(65,66)^. It is important to note that the age at which adults first consumed ED is directly related to the introduction of these drinks to the European market. Since ED were introduced in the late 1980s, adults’ first consumption occurred later compared to the first consumption in teenagers today^(5)^. The age of first consumption being at such a young age is worrying, especially because the adverse effects are greater in children. The risk of intoxication in children is higher due to the fact that they are still growing and their lack of pharmacological tolerance^(79)^. Some studies like Chuda et al.^(49)^ and Casuccio et al.^(60)^ investigated, along with the prevalence of consumption, the adverse effects suffered. In both studies, more than 20 % of those who consumed ED suffered disorders related to these beverages. Casuccio et al.^(60)^ associated a greater probability of suffering these effects with being female.

Some studies have stated that knowing about the effects of ED may decrease their consumption^(60,80)^. Most people have a low perception of the risk of ED^(57)^, with this perception being lower in men^(81)^. This low perception of risk makes ED a gateway for other drugs. This theory suggests that caffeine may lead to the use of other legal drugs, including alcohol and nicotine^(5,10,82)^, which would explain the association of a higher consumption of ED with both alcohol and tobacco.

It is widely observed that health education helps reduce addictive behaviours and improve global health^(83)^. On the other hand, raising public awareness is also important to design policies that protect the population. For example, Poland, which is one of the countries with the highest number of studies carried out, prohibited sales to minors under 16 years of age in 2023^(19)^. Other countries such as Latvia^(84)^ have also taken this measure and, in addition, this country has established advertising restrictions and higher taxes for ED. Other measures applied to ED are maximum caffeine limits, as they have done, for example, in Denmark^(85)^. In the European Union, it is mandatory that ED display the following warning on the label: ‘High caffeine content. Not recommended for children or pregnant or breast-feeding women’^(86)^. Given the high consumption in European countries and the lack of regulatory measures in the majority of countries, legislative measures should be tightened to protect the most vulnerable populations.

Regarding the association of ED consumption with socio-economic-related variables, numerous previous studies have related lower socio-economic status with unhealthy lifestyles and poor health outcomes^(87)^. There is an even greater association of ED consumption with obesity^(88)^ than with unhealthy and poor health outcomes due to its high sugar content, in addition to the association with lower socio-economic status.

Some discrepancies in consumer characteristics between studies could be due to the difference in the frequency of consumption with which they are associated. For example, high educational level is associated with lifetime use of ED^(64)^, while a low educational level is associated with a higher frequency of consumption (weekly^(9)^ or daily^(44)^).

### Limitations and strengths of the study

This review has some limitations. High variability in definition and frequency of consumption prevented any conclusions about the true prevalence of ED consumption in the populations. After performing a meta-regression including those studies that estimated daily ED consumption (data not shown), heterogeneity was estimated at 100 %. This value made it impossible to obtain reliable meta-analysed prevalences. Five studies identified in the review were not described because they did not provide information about geographical location. In addition, some prevalences were not taken into account because of the lack of frequency data. Self-reported questionnaires are a valid method to obtain information about risk factors; however, interviewer-conducted questionnaires (face-to-face or Computer Assisted Telephone Interview (CATI)) improve data quality. In this review, most of the studies were rated as low quality, with the method of obtaining information being one of the most challenging factors. Also, not all European countries had studies that estimated the prevalence of ED consumption.

This review also has strengths. The search strategy was not limited by country. Therefore, as a first step, all studies conducted worldwide were identified, and those focusing on European countries were selected in a subsequent step. Moreover, the search was very exhaustive. We performed a bibliographical search in four databases (MEDLINE, EMBASE, Scopus and Cochrane), in contrast to the two databases of a previous study with similar characteristics (Aonso-Diego et al.)^(69)^. In addition, all references to the included studies were thoroughly reviewed. Inclusion and exclusion criteria were more detailed than Aonso-Diego et al. including special populations, the amount of data collected and the inclusion of the characterisation of consumers. This is the first study to review the characterisation of ED consumers. Data extraction was done through peers.

Studies that estimated the prevalence of ED consumption are highly different, even for the same frequency, population and country, and have low quality, exposing flaws in their methodology. The most studied frequency is weekly consumption, and the most studied population is students. Those who assessed consumption over time found an increase in ED consumption. Those who characterised consumers varied in the characteristics ascertained, being socio-economic-related variables, alcohol consumption, physical activity, tobacco use, age and sex the most studied. Higher consumption is related to older school students and younger university students, lower socio-economic status and substance use such as tobacco and alcohol. Higher consumption is also related to males who, with athletes, are the main target of marketing campaigns.

Given the health problems that have been related to ED consumption, regulation of these beverages is essential, especially in youth. Similar regulations to those for high-fat products or their prohibition of their sale to minors could be effective measures to prevent consumption by young people, who are the most affected population.

## Supporting information

Teijeiro et al. supplementary material Teijeiro et al. supplementary material

## Supplementary material

For supplementary material accompanying this paper visit https://doi.org/10.1017/S1368980025100463
