# Supplementary material for: Prevalence and characterisation of energy drink consumption in Europe: a systematic review
Source: Public Health Nutr. 2025 Jun 3;28(1):e119. doi: 10.1017/S1368980025100463 (PMC12465085; doi:10.1017/S1368980025100463)
Supplement: Teijeiro et al. supplementary material [file S1368980025100463sup001.docx]

**APPENDIX A**

**Supplementary Table 1.** Search strategy

| **BASE DE DATOS** | **Consulta** | **TOTAL RECORDS** |
| --- | --- | --- |
| EMBASE | (exp "Energy Drinks"/ OR ("energy drink" or "energy drinks").ab,hw,ti. OR ((caffein* or taurin*).ab,hw,ti. AND (drink or drinks or beverage or beverages).ab,hw,ti.)) AND (Consumer or consumers or consumption).ab,hw,ti. AND ((exp Prevalence/ or exp Epidemiology/) OR (prevalence or epidemiology).ab,hw,ti. | 961 |
| Medline (Ovid) | (exp "Energy Drinks"/ OR ("energy drink" or "energy drinks").ab,hw,ti. OR ((caffein* or taurin*).ab,hw,ti. AND (drink or drinks or beverage or beverages).ab,hw,ti.)) AND (Consumer or consumers or consumption).ab,hw,ti. AND ((exp Prevalence/ or exp Epidemiology/) OR (prevalence or epidemiology).ab,hw,ti. | 282 |
| Scopus | ( ( INDEXTERMS ( "Energy Drinks" ) ) OR ( ( TITLE-ABS ( "energy drink" ) OR TITLE-ABS ( "energy drinks" ) OR ( TITLE-ABS ( caffein* ) OR TITLE-ABS ( taurin* ) ) AND ( TITLE-ABS ( drink* ) OR TITLE-ABS ( beverag* ) ) ) ) ) AND ( TITLE-ABS ( consumer ) OR TITLE-ABS ( consumers ) OR TITLE-ABS ( consumption ) ) AND ( TITLE-ABS ( prevalence ) OR TITLE-ABS ( epidemiology ) OR INDEXTERMS ( prevalence ) OR INDEXTERMS ( epidemiology ) ) | 541 |
| Cochrane | ("energy drink" OR "energy drinks" OR ((caffein* OR taurin*) AND (drink* OR beverag*))):ti,ab,kw AND (Consumer OR consumers OR consumption):ti,ab,kw AND (prevalence OR epidemiology):ti,ab,kw | 100 |
| TOTAL |  | 1884 |

**Supplementary Table 2.** Adapted Newcastle-Ottawa scale

| **SECTION 1: SELECTION OF THE SAMPLE (max 4****)** | |
| --- | --- |
| **Representativeness of the sample** |  |
| a) Truly representative of the target population (all subjects or it is indicated that the sample is representative and there is random sampling of the population) | ** |
| b) Somewhat representative of the target population (non-random sampling) | * |
| c) No description of the sampling strategy or recruitment |  |
|  |  |
| **Sample size** |  |
| a) Justified | * |
| b) Not justified |  |
|  |  |
| **Renspondents vs. Non-respondents** |  |
| a) The response rate is satisfactory (≥ 80%) | * |
| b) The response rate is unsatisfactory (< 80%) |  |
| c) No description of the response rate |  |
|  |  |
| **SECTION 2: ASSESSMENT OF ENERGY DRINKS CONSUMPTION (max. 8********)** | |
| **Ascertainment of the consumption** |  |
| a) Face-to-face questionnaire by a trained interviewer | *** |
| b) Face-to-face questionnaire by an interviewer without specifying training or experience | ** |
| c) Self-declared questionnaire (via phone) or self-administered in the presence of a trained or expert interviewer | * |
| d) Self-completed questionnaire (online or electronic submission, through mail, or in printed form) |  |
| e) No description of the measurement tool |  |
|  |  |
| **Definition of the consumption** |  |
| a) The study clearly defines energy drink consumption using the precise wording from the questionnaire | ** |
| b) The study does not define energy drink consumption using the precise wording from the questionnaire | * |
| c) The study does not include a definition of energy drinks consumption |  |
|  |  |
| **Characterization of the consumption** |  |
| a) The study indicates the duration, frequency, and intensity of the consumption | *** |
| b) The study indicates two aspects of the consumption: duration, frequency, or intensity | ** |
| c) The study indicates solely one aspect of the consumption: duration, frequency, or intensity | * |
| d) The study does not specify duration, frequency or intensity of the consumption |  |
|  |  |
| **SECTION 3: COMPARABILITY (max. 1*)** | |
| a) The association between energy drinks consumption and potential confounders were investigated by multivariable analysis | * |
| b) The association between energy drinks consumption and potential confounders were not investigated by multivariable analysis |  |
|  |  |
| **SECTION 4: OUTCOME (max.3***)** | |
| **Stratification of the prevalence** |  |
| a) The study stratifies the results by frequency of consumption, gender or age groups of the participants, when applicable (at least by one criterion) | * |
| b) The study provides a global prevalence of consumption, regardless of the frequency and characteristics of the participants |  |
|  |  |
| **Statistical test** |  |
| a) The statistical test used to analyze the data is clearly described and appropriate | * |
| b) The statistical test is not appropriate, not described or incomplete. |  |
|  |  |
| **Assessment of potential biases/limitations** |  |
| a) The study assesses the possibility of biases and limitations arising from the design and selection of the sample | * |
| b) The study does not assess the possibility of biases and limitations arising from the design and selection of the sample |  |

**Supplementary Table 3.** Assessment of quality of the studies (Newcasttle-Ottawa)

|  |  | Section 1 | | | Section 2 | | | Section 3 | Section 4 | | |  | |
| --- | --- | --- | --- | --- | --- | --- | --- | --- | --- | --- | --- | --- | --- |
| Author | Year of publication | Representativeness of the sample | Sample size | Renspondents vs. non-respondents | Ascertainment of the consumption | Definition of the consumption | Characterization of the consumption | Comparability | Stratification of the prevalence | Statistical test | Assessment of potencial biases /limitations | Total score | Quality |
| Oteri A | 2007 |  |  | * |  | * | ** |  |  |  |  | 4 | Low |
| Braun H | 2009 |  |  |  |  |  | * |  |  | * |  | 2 | Low |
| Gambon DL | 2011 |  |  |  |  | * | ** |  | * | * | * | 6 | Low |
| James JE | 2011 |  |  | * |  | * | * |  |  | * | * | 5 | Low |
| Abreu AR | 2013 |  |  |  |  | ** | ** |  | * |  |  | 5 | Low |
| Gallimberti L | 2013 |  |  |  | * | ** | * | * | * | * | * | 8 | Low |
| Kristjansson AL | 2013 | * |  |  |  | ** | * |  |  | * | * | 6 | Low |
| Maier LJ | 2013 |  |  |  |  | ** | ** |  | * | * | * | 7 | Low |
| Zucconi S | 2013 | ** | * |  | * | ** | *** |  | * | * | * | 12 | Moderate |
| Friis K | 2014 |  |  |  |  | ** | * | * | * | * | * | 7 | Low |
| Flotta D | 2014 |  |  |  |  | * | * | * | * | * | * | 6 | Low |
| Gornicka M | 2014 |  |  |  |  |  |  |  | * | * |  | 2 | Low |
| Kristjansson AL | 2014 | * |  | * |  | ** | ** |  | * | * | * | 9 | Moderate |
| Rudolph E | 2014 | * |  |  | *** | * | * |  |  | * | * | 8 | Low |
| Chuda A | 2015 |  |  |  |  | ** | ** |  | * | * | * | 7 | Low |
| Vilija M | 2014 |  |  | * | * | ** | * |  |  | * | * | 7 | Low |
| Casuccio A | 2015 |  |  |  |  | ** | ** |  | * | * | * | 7 | Low |
| Gallimberti L | 2015 |  |  |  | * | ** | * |  | * | * | * | 7 | Low |
| Kristjansson AL | 2015 | * |  |  |  | ** | ** |  | * | * | * | 8 | Low |
| Liakoni E | 2015 |  |  |  | * | ** | * |  | * | * | * | 7 | Low |
| Nowak D | 2015 |  |  |  |  | ** | * |  | * | * |  | 5 | Low |
| Cencek P | 2016 |  |  |  |  |  | * |  | * | * |  | 3 | Low |
| Barrense-Dias Y | 2016 | * |  |  |  | ** | * | * | * | * | * | 8 | Low |
| Koivusilta L | 2016 |  |  |  |  | ** | * |  | * | * | * | 6 | Low |
| Milovanovic DD | 2016 |  |  |  |  |  | * |  | * | * | * | 4 | Low |
| Pacifici R | 2016 |  | * | * |  | * | * |  | * | * | * | 7 | Low |
| Parezanovic GS | 2016 |  |  |  |  |  | * |  | * | * |  | 3 | Low |
| Richards G | 2016 |  | * |  |  | ** | * |  | * | * | * | 7 | Low |
| Treur JL | 2016 |  | * |  |  | * | * |  | * | * | * | 6 | Low |
| Vitiello V | 2016 |  |  | * |  |  | * |  | * | * | * | 5 | Low |
| Wardenaar F | 2016 | * |  |  |  | ** | * |  | * | * | * | 7 | Low |
| Wierzejska R | 2016 |  |  |  | ** | * | * |  | * | * | * | 7 | Low |
| Casuccio A | 2017 |  |  |  | *** | ** | ** |  | * | * |  | 9 | Moderate |
| Concerto C | 2017 |  |  | * |  | * | * |  |  | * | * | 5 | Low |
| Holubcikova J | 2017 | ** | * | * | * | * | * |  | * | * | * | 10 | Moderate |
| Holubcikova J | 2017 | ** | * | * | * | ** | * | * | * | * | * | 12 | Moderate |
| Husarova D | 2017 | ** | * | * | * | ** | * |  |  | * | * | 10 | Moderate |
| Scalese M | 2017 | * |  | * |  | * | * | * | * | * | * | 9 | Moderate |
| Buja A | 2018 |  |  |  | * | ** |  |  |  | * | * | 5 | Low |
| Degirmenci N | 2018 |  | * |  |  | ** | ** | * | * | * | * | 9 | Moderate |
| Majori S | 2018 | * |  | * |  | ** | ** | * | * | * | * | 10 | Moderate |
| Martins A | 2018 |  |  |  |  | ** | *** |  | * |  | * | 7 | Low |
| Scuri S | 2018 |  |  |  |  |  |  |  |  |  |  | 0 | Low |
| Thomas F | 2018 | * |  |  |  | ** |  |  |  | * | * | 5 | Low |
| Totaro M | 2018 |  |  |  |  |  | *** |  | * | * |  | 5 | Low |
| Galimov A | 2019 | * |  |  | * | ** | ** | * | * | * | * | 10 | Moderate |
| Gruzieva, TS | 2019 |  |  |  |  |  | ** |  |  |  |  | 2 | Low |
| Benkert R | 2020 | ** | * |  |  | ** | * | * | * | * | * | 10 | Moderate |
| Buja, A | 2020 | ** |  | * |  | ** | * |  |  | * | * | 8 | Low |
| Cruz Munoz V | 2020 | * |  |  | * | ** | ** |  | * | * | * | 9 | Moderate |
| Holguín EP | 2020 |  |  | * |  |  | * |  | * | * | * | 5 | Low |
| Lebacq T | 2020 | ** |  |  |  | ** | * | * | * |  | * | 8 | Low |
| Lehmann F | 2020 | ** |  |  | *** |  | * |  | * |  |  | 7 | Low |
| Sljivo A | 2020 |  | * |  |  | * | *** |  | * | * | * | 8 | Low |
| Tanner T | 2020 | * |  | * |  | ** | * |  |  | * | * | 7 | Low |
| Toth A | 2020 | * |  |  |  | * | * |  | * | * | * | 6 | Low |
| Błaszczyk-Bębenek E | 2021 |  |  |  |  | * | * |  |  | * |  | 3 | Low |
| Boleslawska I | 2021 |  |  |  |  | * | ** |  | * | * | * | 6 | Low |
| Brumboiu I | 2021 |  |  |  |  | * | ** |  |  | * | * | 5 | Low |
| Dąbrowska-Galas M | 2021 |  | * |  | * | * | * |  | * | * | * | 7 | Low |
| Fernandes | 2021 |  |  |  |  |  |  |  | * | * |  | 2 | Low |
| Franke AG | 2021 |  |  |  |  | * | * |  | * | * | * | 5 | Low |
| Halldorsson TI | 2021 | * |  |  | * | * | ** |  | * | * |  | 7 | Low |
| Jebrini T | 2021 | * |  |  |  | * | * |  | * | * | * | 6 | Low |
| Kaldenbach S | 2021 |  |  | * |  | ** | * | * | * | * | * | 8 | Low |
| Morgan K | 2021 |  |  |  |  | ** | * | * | * | * | * | 7 | Low |
| Oliver Angles A | 2021 | ** | * |  |  | ** | * | * | * | * | * | 7 | Low |
| Puupponen, M | 2021 | ** |  |  |  | ** | * | * | * | * | * | 9 | Moderate |
| Scalese. M | 2021 | ** |  | * |  | ** | * |  | * | * | * | 9 | Moderate |
| Schroder H | 2021 |  |  |  |  | ** | ** | * | * | * | * | 8 | Low |
| Svensson A | 2021 |  |  |  |  | ** | * | * | * | * | * | 7 | Low |
| Vasic J | 2021 |  |  |  | * |  |  |  |  | * | * | 3 | Low |
| Atienza-Carbonell B | 2022 |  |  |  |  |  |  |  |  | * | * | 2 | Low |
| Brunborg GS | 2022 |  |  | * |  | ** | * | * |  | * | * | 7 | Low |
| Jeannou B | 2022 | ** |  |  |  |  |  |  |  | * | * | 4 | Low |
| Kaldenbach S | 2022 | * |  | * |  | ** | * |  |  | * | * | 7 | Low |
| Kosendiak AA | 2022 |  |  |  |  | ** | * |  |  |  | * | 4 | Low |
| Riera-Sampol A | 2022 |  |  |  |  | ** | * |  | * | * | * | 6 | Low |
| Sammito S | 2022 |  |  |  |  |  |  |  |  |  | * | 1 | Low |
| Soukiasian PD | 2022 | ** |  |  | *** |  |  |  | * | * |  | 7 | Low |
| Tomanic M | 2022 | * |  |  |  | ** | * | * | * | * | * | 8 | Low |
| Pavlovic N | 2023 |  |  |  |  | * | ** |  | * | * | * | 6 | Low |
| Vogel C | 2023 | ** |  |  | ** | * | * | * | * | * |  | 9 | Moderate |

**Supplementary Table 4.** Summary of global energy drink consumption prevalence by study year, population group, and European country

|  | Overall prevalence range (%) | Monthly prevalence range (%) |
| --- | --- | --- |
| Study year | |  |
| 2007 | 14.8 | - |
| 2009 | 25.0 | - |
| 2011 | 38-39.4 | - |
| 2013 | 1.3-85.0 | 6.5-29.7 |
| 2014 | 1.5-67.7 | 21.4 |
| 2015 | 2.1-75.4 | 20.0-30.6 |
| 2016 | 1.0-78.5 | 19.8 |
| 2017 | 3.7-77.9 | 50.9 |
| 2018 | 12.0-56.7 | 14.7-34.0 |
| 2019 | 21.4-61.7 | 21.4 |
| 2020 | 6.1-83.9 | 8.9-49.2 |
| 2021 | 1.3-62.8 | 8.3-31.6 |
| 2022 | 0.5-50.0 | 13.0-29.0 |
| 2023 | 2.1-70.3 | 4.6-52.4 |
| 2024 | 2.7-39.9 | 18.1 |
| Population group | |  |
| Adults | 1.3-77.9 | 4.6-50.9 |
| University students | 0.5-83.9 | 6.3-52.4 |
| Adolescents | 48.0-85.0 | - |
| School students | 1.0-75.4 | 6.5-49.2 |
| Children | 6.0-40.0 | - |
| Country | |  |
| Austria | 9.0-75.0 | - |
| Belgium | 8.0-85.0 | - |
| Bosnia | 61.7 | - |
| Croacia | 12.6-52.4 | 52.4 |
| Cyprus | 11.0-53.0 | - |
| Czech Republic | 40.0-82.0 | - |
| Denmark | 15.8 | - |
| Finland | 2.8-65.0 | - |
| France | 8.2-66.0 | - |
| Germany | 8.9-62.8 | 8.9-31.6 |
| Greece | 10.0-48.0 | - |
| Hungary | 2.7-78.0 | - |
| Iceland | 20.2-56.0 | - |
| Italy | 1.3-77.9 | 6.3-50.9 |
| Lithuania | 21.0-34.8 | - |
| Norway | 1.0-54.4 | 28.0 |
| Poland | 0.5-78.5 | 8.3-29.0 |
| Portugal | 14.1-56.7 | 34.0 |
| Romania | 4.6-70.0 | 4.6 |
| Serbia | 1.0-19.3 | - |
| Slovakia | 1.6-36.7 | - |
| Spain | 1.3-83.9 | 13.0-49.2 |
| Sweeden | 2.3-69.0 | - |
| Switzerland | 6.1-75.4 | 24.6-29.7 |
| The Netherlands | 5.8-67.0 | - |
| Ukraine | 32.5 | - |
| United Kingdom | 6.0-69.0 | 30.6 |
